# Supplementary material for: Effects of stochastic coding on olfactory discrimination in flies and mice
Source: PLoS Biol. 2023 Oct 31;21(10):e3002206. doi: 10.1371/journal.pbio.3002206 (PMC10618007; doi:10.1371/journal.pbio.3002206)
Supplement: S1 Text — Fig B. Responses in Mouse PCx cells are more variable compared to flies. Fig C. Reliable cells preserve odor-similarity better than unreliable cells. Fig D. Unreliable cells are more likely to respond differently between similar odors compared to reliable cells. Fig E. The probability of overlap cell increases from similar to dissimilar odors. Fig F. Unreliable cells are a composition of cells with different levels of reliabilities. Fig G. Noise in the winner-take-all mechanism produces a stochastic code. Fig H. Sparse coding does not improve discrimination ability for similar odors. Fig I. The effect of the significance levels on discrimination analysis. Fig J. The effect of the significance levels on discrimination analysis. Fig K. Fly circuit model parameter explorations. Table A. Possible contributions by cell x towards discrimination. Table B. The top 40 parameter combinations (out of 44,217) of the single synapse model that produced results most similar to the ones observed in Fig 1C and 1D. Table C. Results of the performance of 3 linear classifiers/decoders on the fly and mouse datasets. Table D. The response characteristics (Fig 1) for all the flies that were examined. Table E. The response characteristics (Fig 1) for all the mice that were examined. (PDF) [file pbio.3002206.s001.pdf]

Effects of stochastic coding on olfactory discrimination in flies and mice: Supplement

This document contains supplementary figures, tables, and methods.

Supplementary Figures

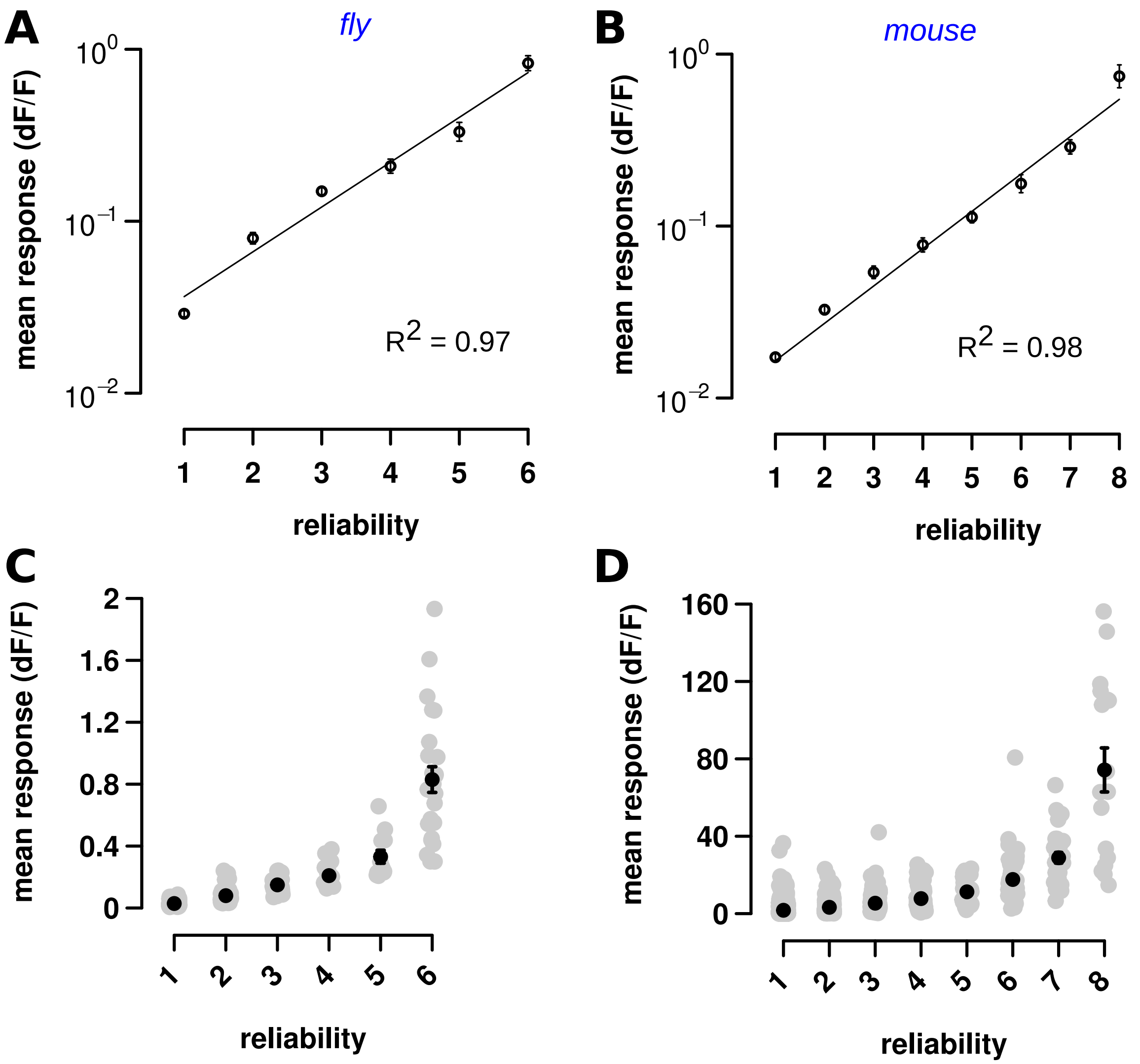

Figure A: **Odor response sizes in the fly MB and mouse PCx cells increases with reliability**. (A-D) Mean response size of each cell plotted against its reliability level (on the x-axis) for fly (A,C) and mouse (B,D). (A,B) Shows the fits for the curves in Figures 1E,H. In both cases the exponential equation $y = ae^{bx}$ has been converted to the form $\log(y) = \log(a) + bx$ in order to do a least squares linear regression fit, with the fit r shown in the plots. Error bars are mean $\pm$ SEM, and have been converted to a log scale taking care that the log error is properly propagated. (C,D) The two figures are similar to Figures 1E,H, except here all the points are plotted instead of just the means. Each grey circle denotes a cell's response to an odor on the x-axis, and represents the mean response size of that cell on the y-axis. The reliability number on the x-axis denotes the number of trials (reliability) for which that cell had significant responses. Error bars are mean $\pm$ SEM. Plots A,B and C,D use the fly main dataset on Zenodo and mouse dataset 164 on Dandi, respectively. The links for the datasets for this and other supplementary figures are in the Data Availability section. The data underlying the graphs shown in the figure can be found in S7 Data.

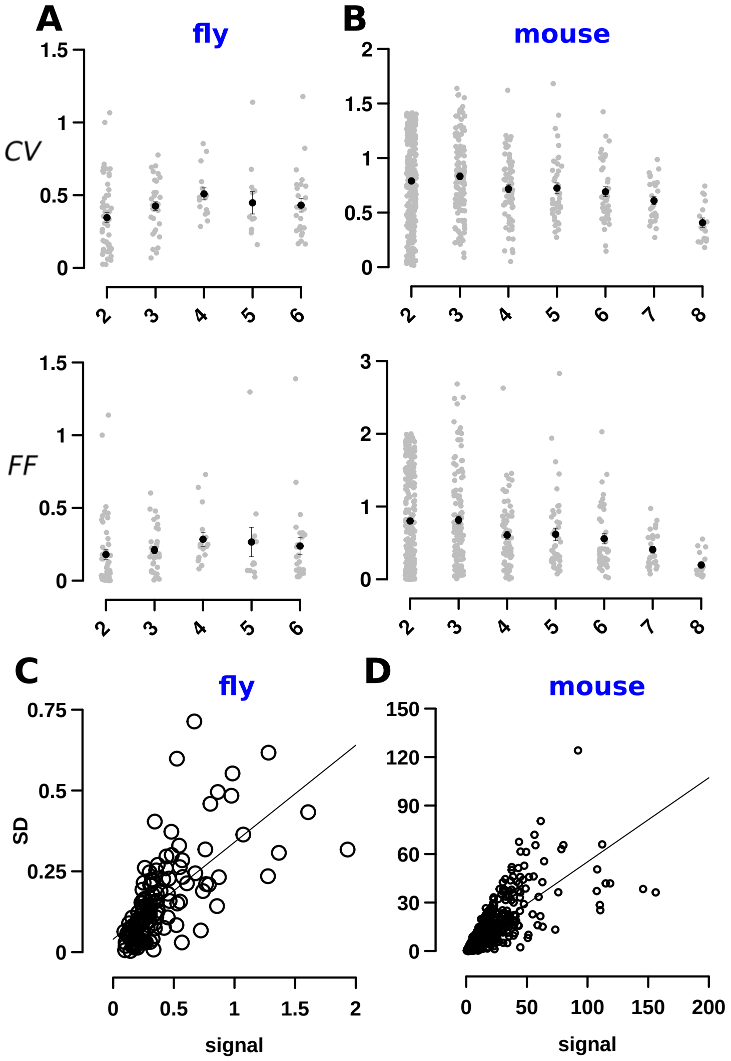

Figure B: **Responses in Mouse PCx cells are more variable compared to flies**. (A) Coefficient of Variation (CV) and fano factor (FF) of cell responses in flies. (A, top) shows CV (on the y-axis) with cells split according to their reliability (on the x-axis). The plot does not include cells with a reliability of 1. There is no significant difference in CV across different reliabilities. Error bars in this and all the plots are SEM. (A, bottom) FF for the fly showing how FF differs depending on cell reliabilities. FF for the different cell reliabilities are not significantly different. (B, top) CV for the mouse plotted for different cell reliabilities. CV is greater for the mouse compared to the fly, and decreases with lower cell reliabilities. (B, bottom) FF for the mouse plotted against cell reliabilities shows that FF is higher in the mouse than fly, and FF decreases with cell reliabilities. (C,D) Plot showing how the standard deviation (SD) varies with response magnitude. In both, flies, and mice, SD increases with increase in signal, and are fit for lines with slopes of 0.52 and 0.5. The units are a normalized readout of Ca^{2+} based flourescent activity. Plots A,C and B,D use the fly main dataset on Zenodo and mouse dataset 164 on Dandi, respectively. The data underlying the graphs shown in the figure can be found in S8 Data.

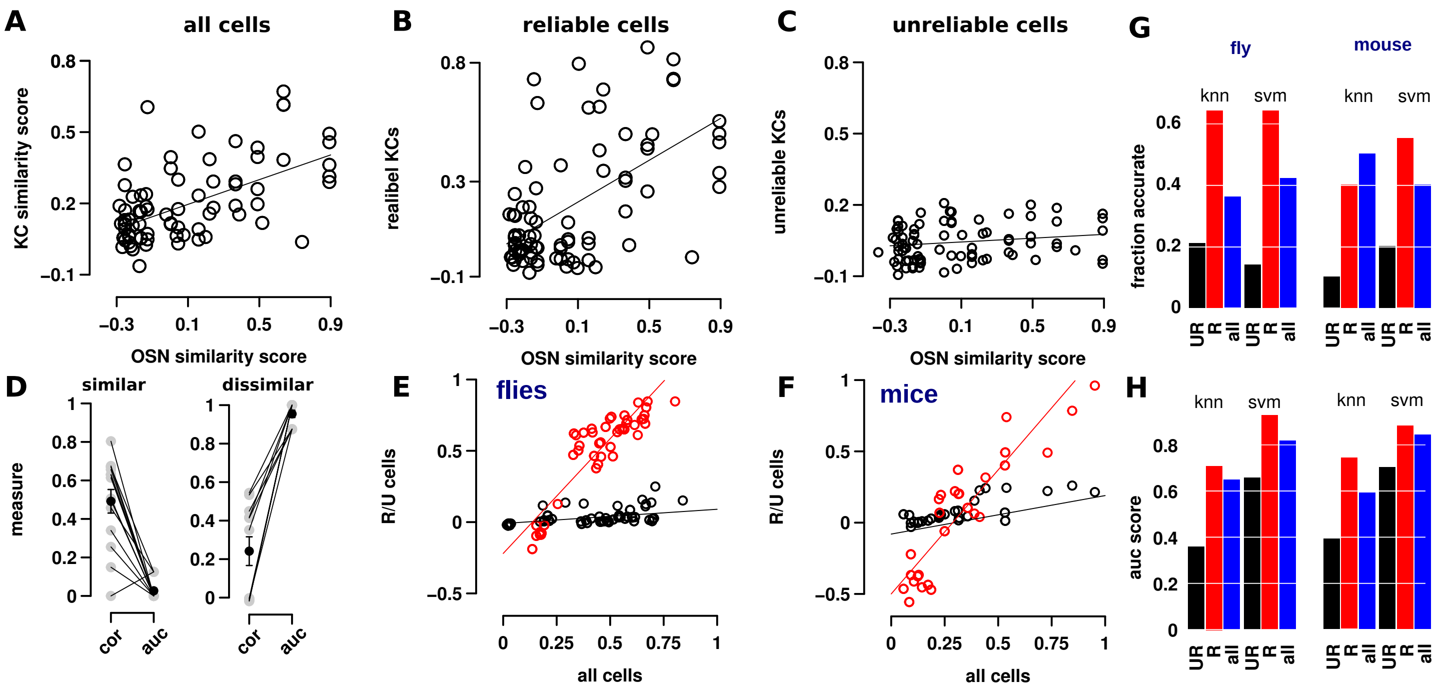

Figure C: **Reliable cells preserve odor-similarity better than unreliable cells**. (A) The similarity between all odor pairs in the fly's nose [1] (x-axis) and MB (y-axis) fits the line fit y = 0.17 + 0.44x (R^2=0.57). Similarity is measured as the correlation between odor-pairs, and each circle denotes one odor-pair. (B) The similarity between odors pair responses in the fly's nose and MB judged by reliable cells alone is a fit for the line fit y = 0.16 + 0.32x (r=0.5). On the other hand, the correlation between fly's nose and MB is low when judged by unreliable cells alone in (C). The line fit has a slope close to 0 (y = 0.04 + 0.04x, r=0.2), though showing a slight trend, wherein odors that are dissimilar (to the left of the x-axis) have less correlation than odors that are similar (to the right of the x-axis). This figure shows similarities for all odor pairs in the fly's antenna and MB, unlike the main figure (Fig. 4) which is restricted to those odor pairs for which correlations in the antenna and MB are within 0.2 of each other. (D) AUC measures the degree to which odors are distinguishable. Comparison of the AUC measure with correlations. In the left panel, are all odor-pairs that have an AUC measure below 0.25 (mean: 0.04) have a corresponding odor similarity of 0.5 (mean) as measured by Pearson's correlation. In the right panel, odor-pairs that have an AUC measure above 0.75 (mean: 0.93) have a corresponding odor similarity of 0.24. (E,F) Similarity of odor-pairs based on all cell populations versus just the population of (R)eliable (in red) or (U)nrelibale (black) cells for both flies and mice. In both species, while Reliable cell similarity is proportional to odor–pair similarity of all cells, the Unreliable cell similarity remains low for all odor–pairs. (G,H) Similar to Fig. 5E for Support Vector Machines (SVM) with a linear kernel and k-Nearest Neighbors (kNN). The plots show these algorithms applied to reliable, unreliable, and all cells. Like LDA, SVM and kNN show that reliable cells are significantly better at classification than unreliable cells. Compare the length of the red bars (reliable, R) to the black bars (unreliable, UR), where the length of the bar denotes the fraction of correct classifications. The performance of decoders with all cells (blue bars) is similar to reliable cells, though slightly less. See Methods (Data Analysis) and Table C for algorithm details and parameters for the decoder, and training and test sets. The plots here use multiple datasets available on the Zenodo and Dandi respositories listed in the Data Availability section. All fly and mouse plots use the fly main dataset on Zenodo and mouse dataset 164 on Dandi, respectively. Additionally, plots A-C made use of the following supplementary fly datasets: 09042009, 110108, 110109_1, 110109_2. The data underlying the graphs shown in the figure can be found in S9 Data.

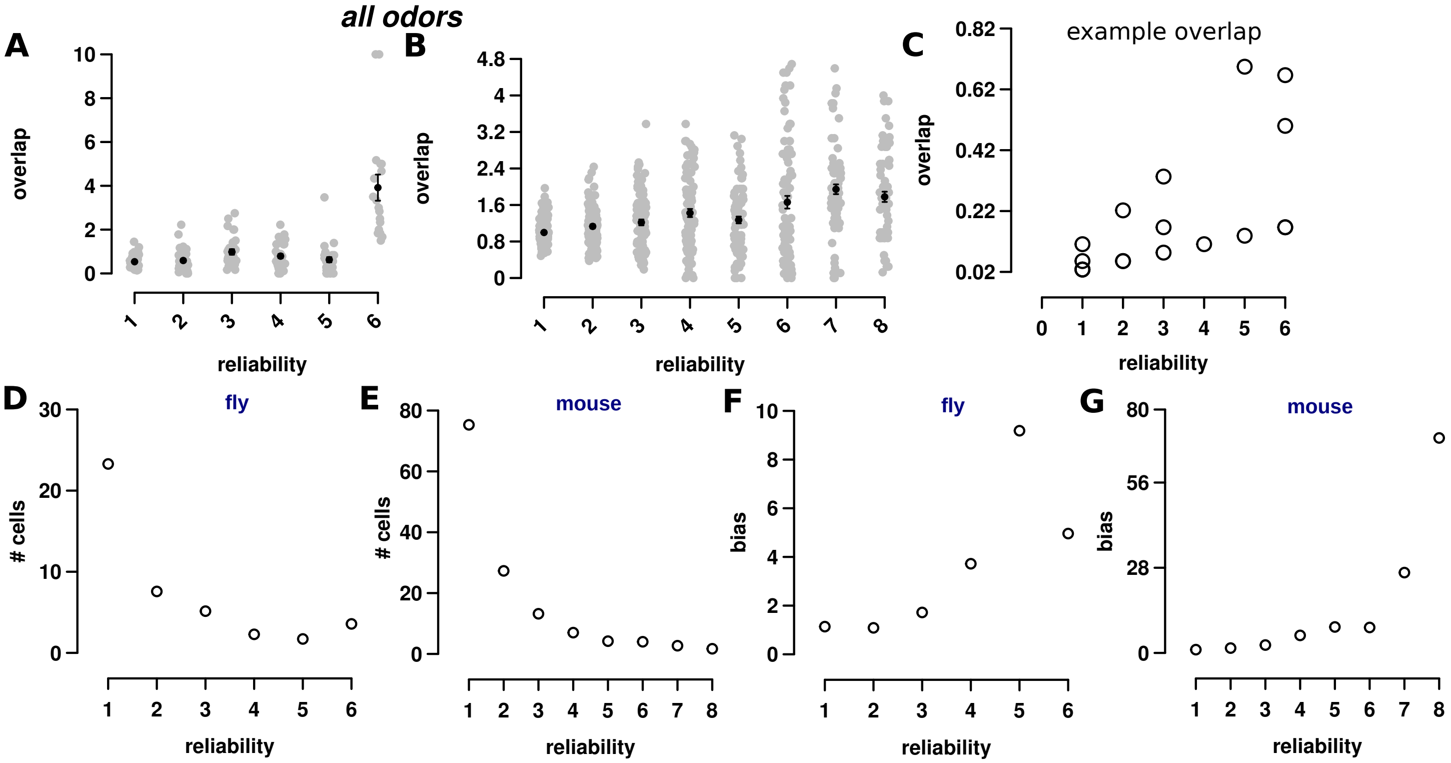

Figure D: **Unreliable cells are more likely to respond differently between similar odors compared to reliable cells**. (A,B) Reliability versus probability of overlap plots for flies (A) and mice (B). For each point or reliability on the x-axis, we plot the corresponding overlap for all odor-pairs while only considering cells with that level of reliability. In both plots, unreliable and reliable cells have similar amounts of overlap, although cumulatively, reliable cells have slightly higher average probability of overlap. (C) An example of using an overlap measure to assess the similarity of odors. As the reliability on the x-axis increases a cell's overlap with the other odor increases. Each circle denotes a single cell and it's overlap measure calculated as the probability of response to odor 1 $*$ probability of response for odor 2. (D,E) Number of cells in each reliability class (cells that all have the same reliability for the odor) for flies and mice. As reliability increases, the number of cells in that reliability class decreases, in both flies (D) and mice (E). (F,G) The bias or selectivity of cells for specific odors in each reliability class decreases as reliability decreases, but is still not random. Each point represents the average bias of cells belonging to a specific reliability class across all odor pairs. Bias is calculated as the measured overlap versus overlap if all cells in that particular group were chosen at random. For instance for odors X and Y, the measured overlap is the number of cells that respond to both odors. The overlap for randomly selected cells is calculated theoretically. It is the expected value of overlap per cell, where the probability of overlap is the product of frequency of the cell class for both odors. Note that for both flies and mice, the bias of reliability 1 cells is greater than 1 ($\approx$ 1.1). The fly datasets in plots A, C, D, and F made use of the fly main dataset on Zenodo, and plots B, E, and G made use of the mouse set 164 on Dandi. The data underlying the graphs shown in the figure can be found in S10 Data.

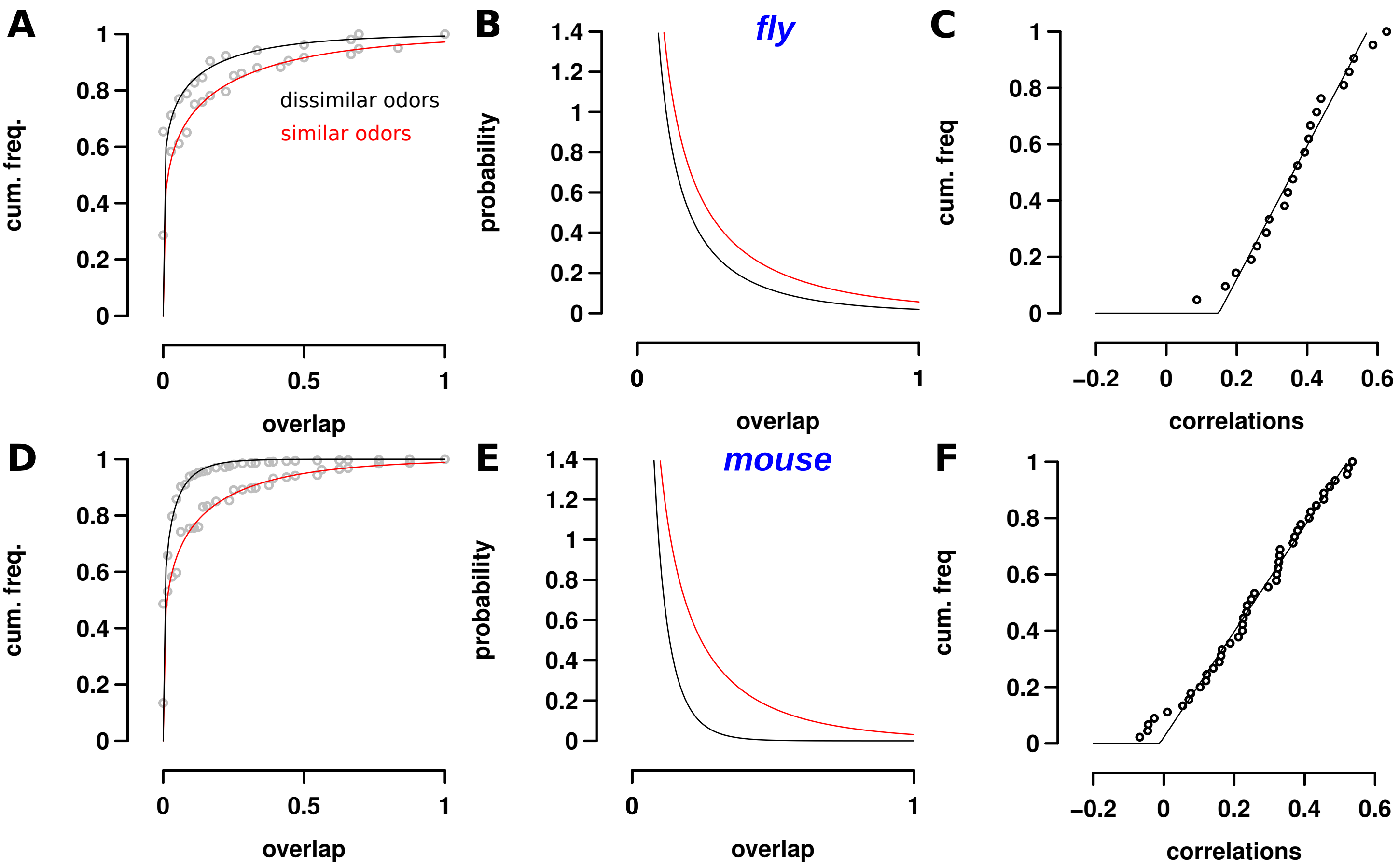

Figure E: **The probability of overlap cell increases from similar to dissimilar odors**. (A-C) Comparison of overlap statistics between similar and dissimilar odors for flies. (A) Shows the cumulative frequency for overlaps between cells for dissimilar (top curve) and similar (bottom curve) odor-pairs. Similar odors were odor-pairs with correlation > 0.5, and dissimilar odor-pairs had correlation < 0.15. The plot shows that for low overlap measures - extreme left - both types of odor-pairs have similar probabilities. But, the probability of higher overlap increases for similar odors. To illustrate the point, we also show the PDF in (B), with similar odor PDFs shown in red. The parameters of the Gamma distribution for are given shape and scale factors of 0.15 and 0.44 for dissimilar and 0.21 and 0.67 for similar odors. (C) The cumulative distribution of correlations between all odor-pairs shows that correlations are uniformly distributed in the interval (0.14,0.57). (D-F) Comparison of overlap statistics between similar (bottom curve in D and red curve in E) and dissimilar odors for mice. The Gamma distribution for mouse is defined by shape and scale factors of 0.24 and 0.09 for dissimilar and 0.22 and 0.45 for similar odors. (D,E) Comparing the overlap distribution for dissimilar and similar odors shows that while the probability of cells having a low overlap is the same, the probability of cells having a high overlap is much higher for similar odors. (F) The similarity between odors is evenly distributed in the dataset that we analyzed. The plot shows the cumulative probability distribution is a good fit for a uniform distribution on the interval (-0.01,0.52). The fly datasets in plots A–C make use of the fly main dataset on Zenodo, and plots D–F make use of the mouse set 164 on Dandi. The data underlying the graphs shown in the figure can be found in S11 Data.

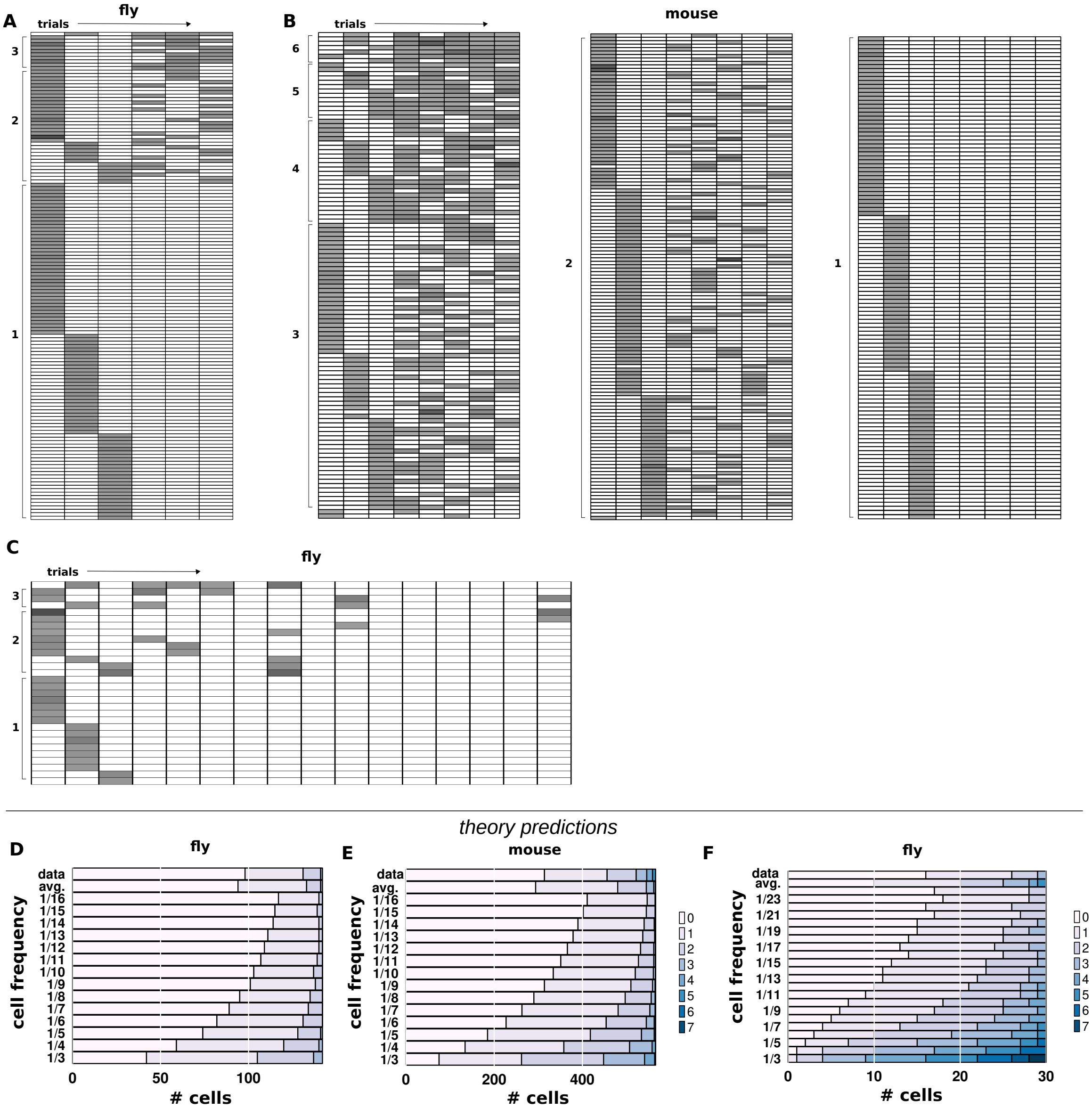

Figure F: **Unreliable cells are a composition of cells with different levels of reliabilities.** (A-C) show stacked plots of responses to odors over 6,8, and 16 trials in the fly and the mouse for cells with only one response in the first 3 trials. Each row shows responses for a cell. In all three cases, cell responses in subsequent trials range over a spectrum of frequencies showing that cell reliabilities likely lie along a continuum. (A) Odor responses of 142 cells to 6 odors across 6 trials in the fly MB. The numbers on the left, in brackets, denote the number of cell responses over 6 trials. The rows are arranged in the decreasing order of number of responses. The rows are also sorted so that cells, e.g., with three responses, with a response in the first trial come first, followed by cells with a response in the second trial, followed by a response in the third trial. (B) and (C) are arranged similarly. (B) Odor responses in 544 cells in the mouse PCx across 8 trials. The last column showing a single response is shortened in the interest of space and represents 245 cells. (C) Odor responses over 16 trials in the fly MB in a total of 30 cells. (D-F) theoretical predictions for the number of cells of each reliability (from trial 4 onwards) for different cell frequencies. (D) Predictions of composition of cell reliabilities in the population for the fly data set with 6 odors and 6 trials per odor for a total of 142 cells. Going from left to right on the x-axis, the shades of blue from light blue to dark blue designate the number of cells that respond with that reliability (legend on right shows the color-frequency mapping). The bottom row shows the prediction if all cells have a base probability of response of 1/3, i.e., they respond once every 3 trials. For a cell population of 142 cells, we would have 42 cells that respond 0 times in trials 4 to 6, 63 cells that respond once, 32 cells that respond twice, and 5 that cells respond 3 times. The designations on the y-axis denote the different cell frequency levels, and the corresponding row, the cell composition. The top two rows show the average of the theory predictions and the data. For the fly, the top two rows are similar suggesting that cells in a population might be composed of different frequencies. (E) Predictions of cell numbers for different cell frequencies for the mouse data set with 10 odors, and 8 trials per odor, for a total of 544 cells. The bottom line/row represents the number of cells at each frequency for trials 4 to 8 if all cells had a base probability of response 1/3. For a cell population of 544 cells, we would have 75 cells that respond 0 times in trials 4 to 8, 187 cells that respond once, 186 cells that respond twice, and 93 cells respond 3 times, 23 cells that respond 4 times, and 2 cells that respond 5 times. The top two rows (like the fly) are similar reflecting that the cell population contains cells with a range base cell response probabilities. (F) Predictions for a fly data set with 1 odor, and 16 trials of the odor for a total of 30 cells. This plot is similar to (D) and (E) with the rows showing cell frequency compositions for cells with different base probabilities. Unlike (D) and (E), the average and data appear dissimilar, although the data does not resemble any particular cell base probability row, showing that it is likely that the cell population is made of a composite of cells with different base probabilities. Plot A used the fly main dataset on Zenodo, plot B used the mouse set 164 on Dandi, and plot C made use of the fly supplementary set 10062010 on Zenodo. The data underlying the graphs shown in the figure can be found in S12 Data.

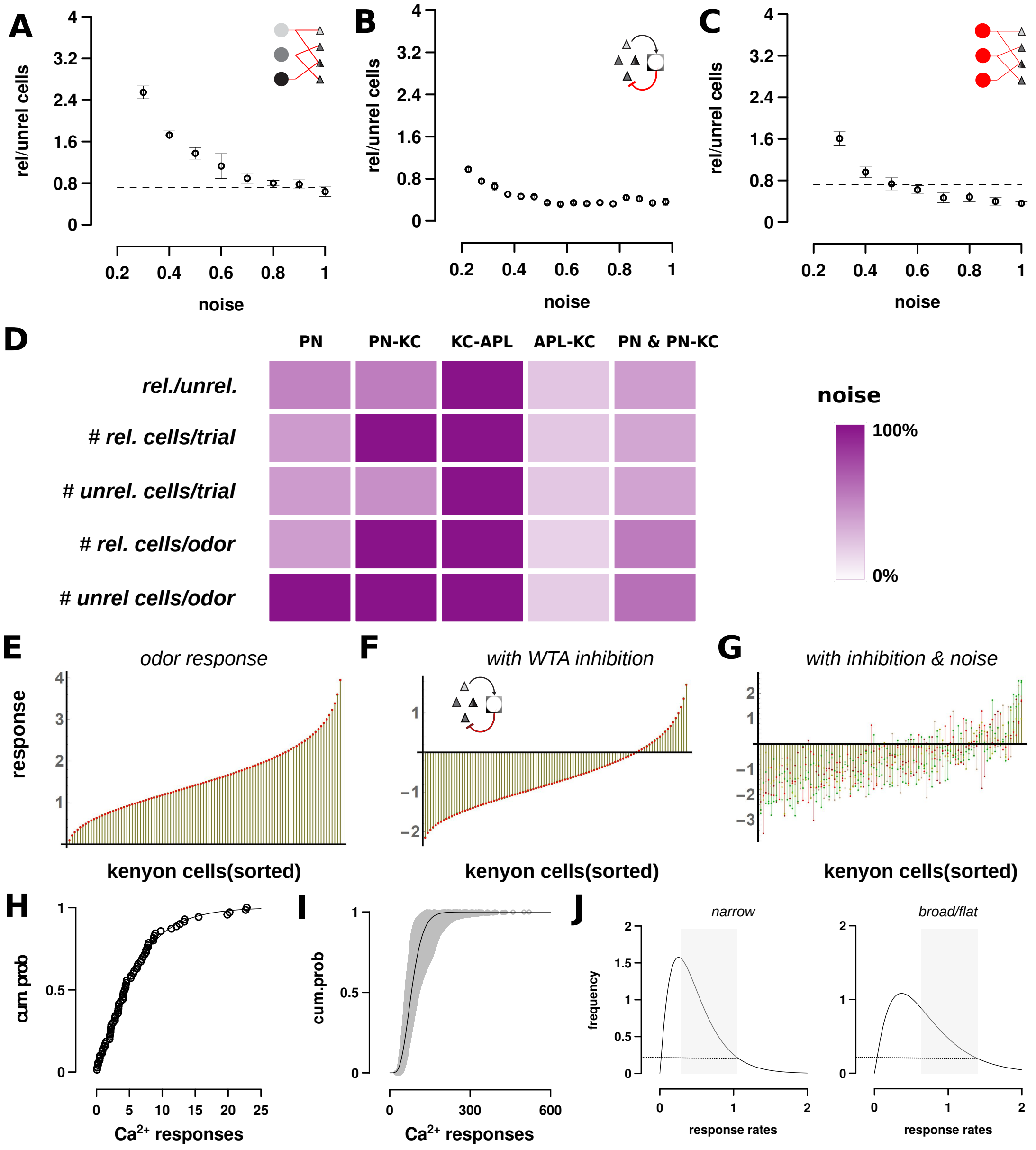

Figure G: **Noise in the winner-take-all mechanism produces a stochastic code**. (A–D) Parameter exploration results for the single synapse model. (A–C) Model simulations show that injecting noise in different parts of the circuit (A) PN→KC synapses, (B) APL-KC synapses, and (C) PNs and PN→KC synapses produces different ratios of reliable to unreliable cells responding per trial. The straight line in the plots denotes reliable/unreliable cell ratio of 0.72 observed with experimental MB responses (Fig. 1). Model (B) requires the least amount of noise (intersection of line and data points at x = 0.275 in F) to observe this ratio. For all plots, the x-axis denotes the fraction of injected noise. Thus, 1 denotes that noise levels are 100% of signal. We show noise levels up to 100%, as the noise levels above it do not produce desired outcomes. (D) Summary of how well each model recapitulated the KC response statistics observed in Figure 1. Each row denotes a response statistic, e.g., the reliable/unreliable ratio. The color intensity indicates the amount of noise required to get response statistics that match data. Darker colors indicate higher amounts of noise. Adding noise to the APL-KC connections produces observed responses with the least amount of noise. For combination model PN & PN-KC, both components are perturbed with the same amount of noise ($>$ 50%); for effects of varying amounts of noise, see Table B and Methods: modeling and theory. (E) A simulation of realistic odor response of KCs without APL inhibition, based on the data from [2]. The response distribution approximates a Gamma distribution and this plot shows KCs on the x-axis sorted in the increasing order of their response. (F) When subjected to APL inhibition fixed to be at 90th percentile of the Gamma distribution, all but the top 10% of cells are silent. The responses are the same from trial to trial as there is no noise. (G) When noise is injected into the feedback, we observe variable responses across all cells with three features. Cells in the top 5% are reliable and their average response is higher. Cells below 50% almost never respond, and cells close to the threshold are highly variable switching between responding and non-responding cells. (D,E) Cumulative frequency plots of odor responses in MB with APL turned off, taken from [2], fit a Gamma distribution with shape=4.12 and scale=5.8. (H) shows an example plot for a single trial, while (I) shows the plot for about 70 different odor trials with the black line showing the (average) fitted Gamma distribution. (J) The breadth of the distribution influences the number of cells affected by noise. Compare the two Gamma distributions with narrow (left, shape = 0.22) and flatter distributions (right, shape = 0.32). The dotted line marks the top 10% of the cells. The width region marked in a shade of grey is equal to the noise amplitude, and shows that the region below the curve is much larger when the distribution is narrow. What this means is that more cells below the cutoff induced by the WTA circuit are influenced by noise, and thus there would be more unreliable cells.

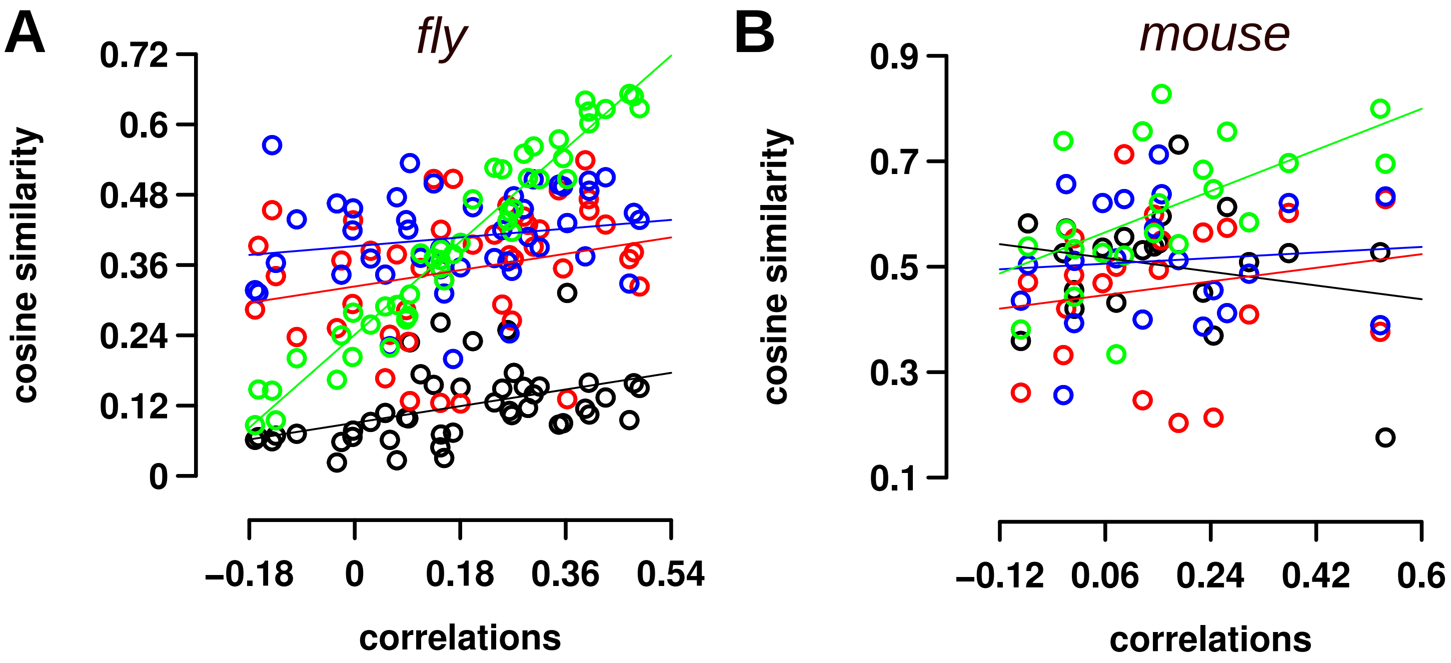

Figure H: **Sparse coding does not improve discrimination ability for similar odors**. (A,B) Comparison of information about odor similarity for different populations of cells in flies and mice. (A) A non-parameterized plot showing how cosine similarity of four groups of cells changes with an increase in odor similiarity (as measured by Pearson's correlation). In green are cells in the top 25% of responding cells, in blue are cells from 25 - 50%, in red are cells from 50 - 75%, and black designates cells in the bottom 25% of responding cells. The slope for cells in green (slope = 0.88) is highest showing that this population best captures the change in odor similarity, similar to reliable cells in Figure 4. It also shows that aggregate (over all odor pairs) similarity is highest for this set of cells. (B) The same plot for mice, showing that the top 25% of cells preserve more odor–similarity information, and that the similarity of odors does not change for the whole population compared to the top 25% of the population. Plot A used the fly main dataset on Zenodo, and plot B used the mouse set 164 on Dandi. The data underlying the graphs shown in the figure can be found in S13 Data.

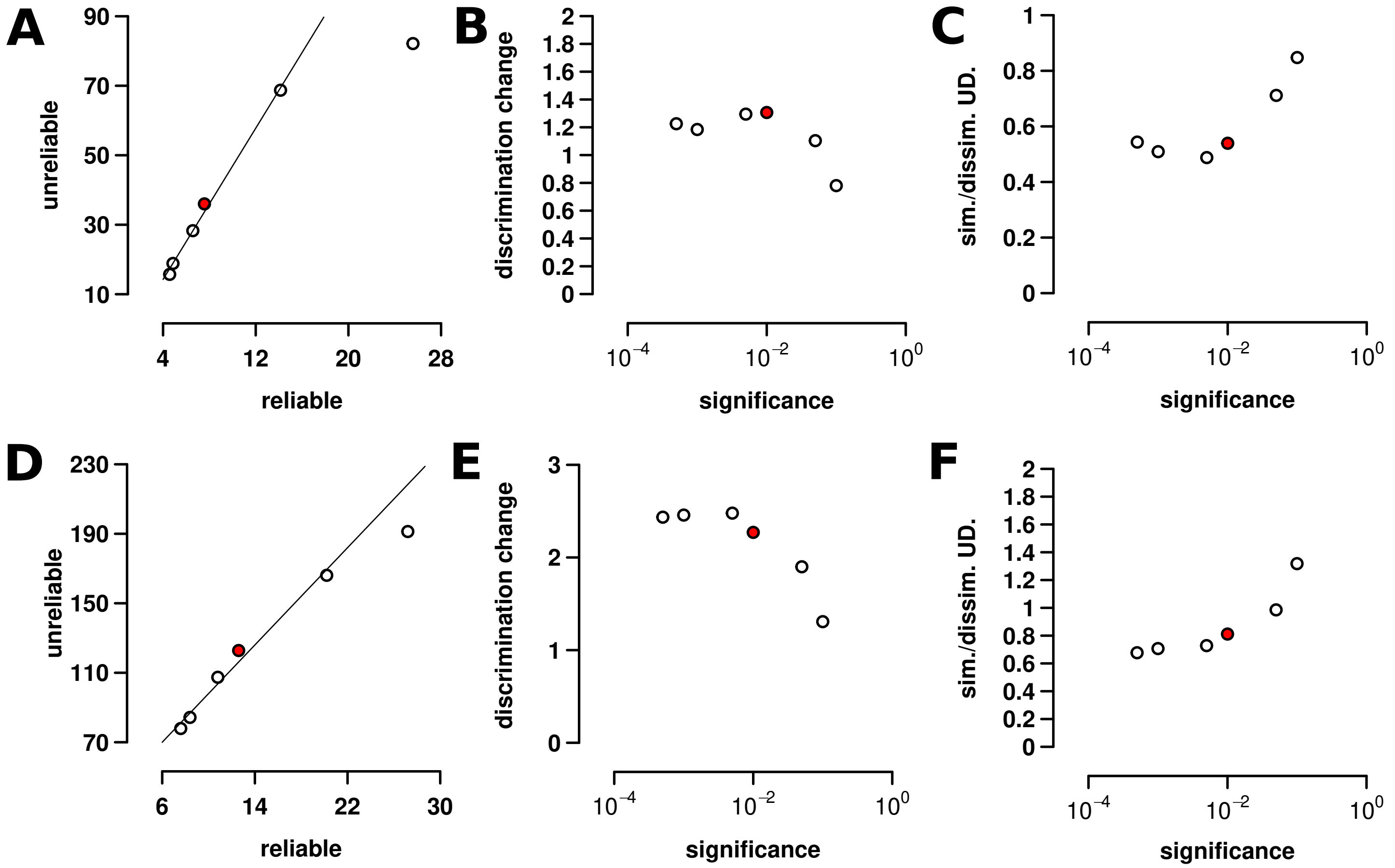

Figure I: **The effect of the significance levels on discrimination analysis.** Effect of significance (p-value) on fly (A–C) and mouse (D–F) results. Note that lowering the p-value increases the significance threshold. In addition to the p-value used in the paper (p = 0.01, 2.33 SDs, red circle in all plots), we examined 5 additional p-values: three lower p-values (0.005, 0.001, and 0.0005, to the left the red circle), and two higher p-values (0.05 and 0.1, to the right of the red circle). (A,D) With an increase in p-values (p = 0.005, 0.01 etc.), the number of responding cells increases, increasing the number of reliable and unreliable cells. For p-value = 0.1, the increase in reliable cells is more than the increase in unreliable cells because there is a finite pool of cells that are available. With a low threshold, most cells become reliable, and there aren't enough cells that can become unreliable to maintain the same reliable/unreliable cell ratio. (B,E) The change in discriminatory ability for similar odors. The y-axis shows the change in discrimination with extended vs. normal training; values larger than 1 indicate a benefit of extended training. (C,F) Discrimination for similar odors normalized to dissimilar odors, with normal training. Values greater than 1 indicate that similar odor discrimination is better. With p-values < 0.05, with few reliable cells and many more unreliable cells in comparison, dissimilar odor discrimination is better. With p-values ≥ 0.05, however, similar odor discrimination is as good as dissimilar odor discrimination. Note that in mice the reliable/unreliable ratio for p-value = 0.1 is closer to the points for other p-values, discrimination change (E) stays above 1 even if there is a similar trend to (B), and similar odor discrimination is better than dissimilar odor discrimination at p-value=0.1 (F). Plots A–C used the fly main dataset on Zenodo, and plots D–F used the mouse set 164 on Dandi. The data underlying the graphs shown in the figure can be found in S14 Data.

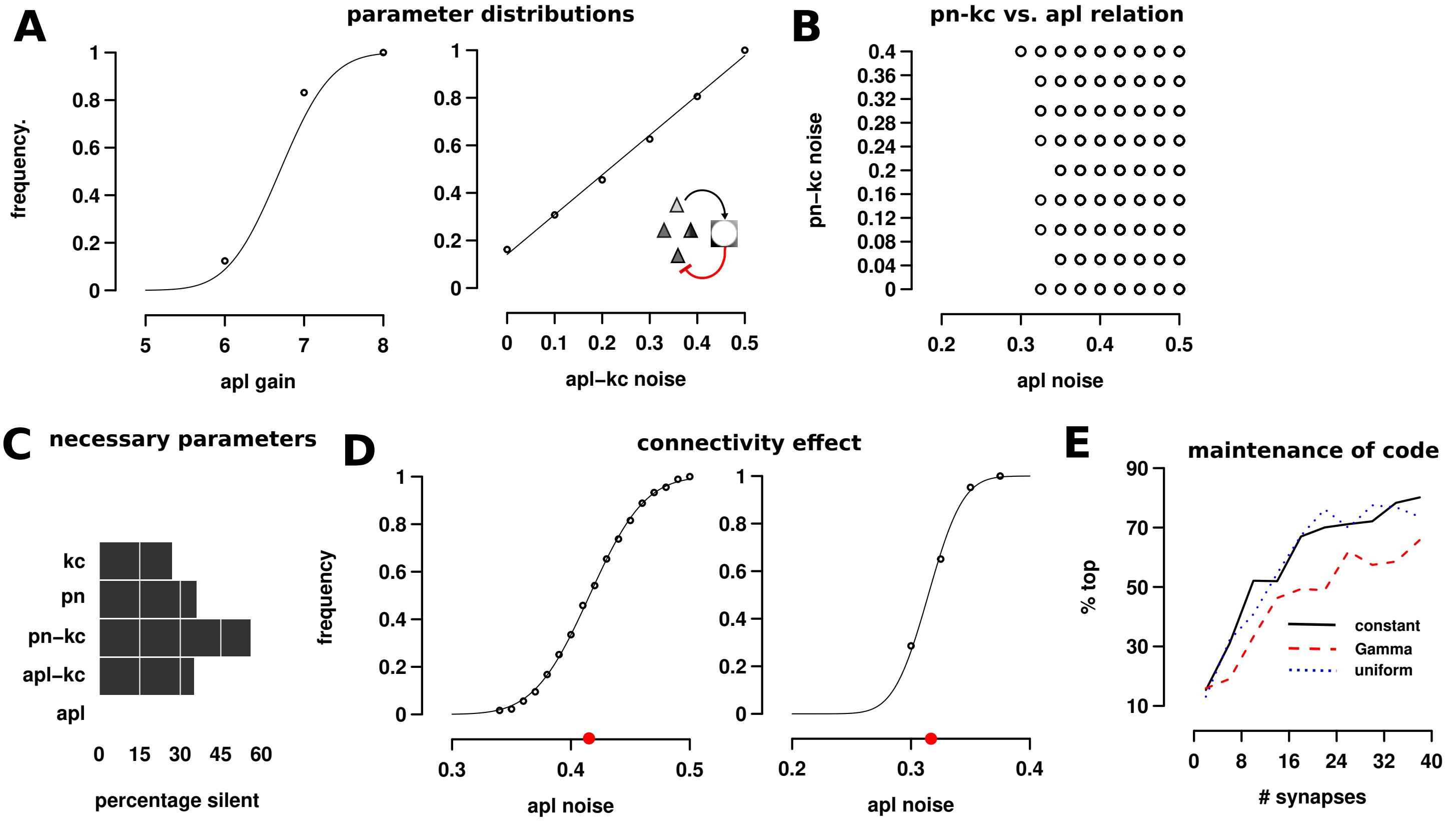

Figure J: **Fly circuit model parameter explorations.** (A) The parameter distribution for APL gain and APL-KC synaptic noise. The APL gain parameter that leads to observed KC stochastic codes is the strength that produces a code with 6-8 cells active. The APL-KC synaptic noise is uniformly distributed. (B) Relationship between PN-KC and APL noise. As the PN-KC value increases, the amount of APL noise needed is the same. (C) Percentage of parameter combinations for which the parameter was not needed. The plot show that the APL was neccessary for observing experimental results. (D) The amount of noise needed in APL to generate successful results is reduced when we move from a Gamma distribution with 6 synapses (left) to a uniform distribution with 20 synapses (right). More synapses means less noise in the the APL inhibition signal to each KC, i.e., to the right of in (E). The red circle indicates mean. (E) Changing the distributions of the APL-KC synapses changes the composition of the top 15 % of the neurons. Here the apl-cutoff was fixed so that, on average, the top 15 % of cells fire. Each line is a different distribution: black: all synapses are the same strength, blue dotted: synapses are generated from a tight (small range) uniform distribution, red dotted: neurons are generated from a Gamma distribution. On the x-axis, you have the mean number of synapses for each APL-KC connection. The number for each APL-KC was generated from a Poisson distribution with this mean. The top % indicates the percentage of cells that remain as top responding neurons. This percentage increases as the number of synapses per APL-KC connection is increased and is also higher for the constant and uniform distributions.

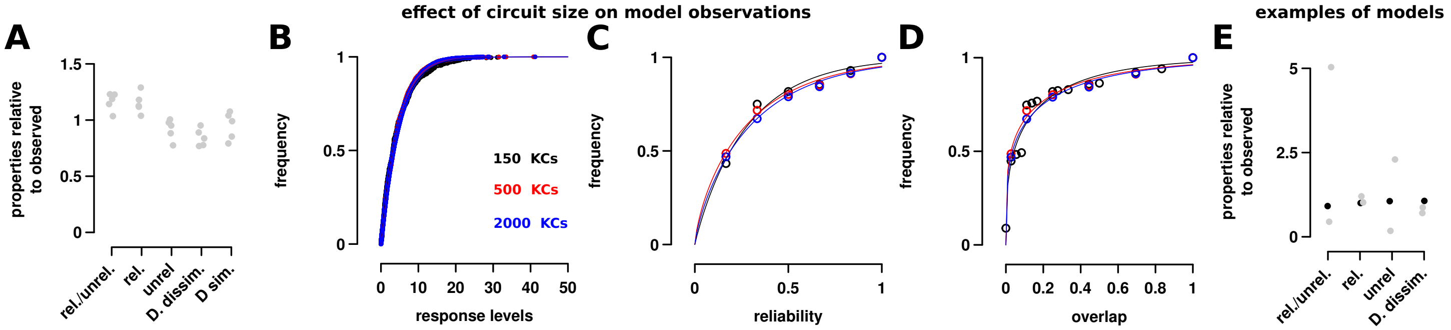

Figure K: **Examples of parameter combinations.** (A-D) The effect of Fly MB size, i.e., number of KCs on the results observed in Figure 1. (A) X-axis shows 5 properties: reliable/unreliable cells, reliable cells per odor, unreliable cells per odor, improvement in discrimination ability for dissimilar and similar odors with extended training. The y-axis shows the values of these properties, normalized to the results in the main paper, for 5 MB sizes: 150, 250, 500, 1000, and 2000 KCs. (B-D) Shows the properties plotted in Figure 2 – cumulative frequency histograms of response levels, reliability, and overlap fitted to Gamma distributions – for for 3 MB sizes: 150, 500, and 2000 KCs. There is no difference in these properties amongst the three sizes, with all them following nearly identical Gamma distributions. (E) Similar figure to (A) except here, we tried three parameter sets, where one set (depicted with a black circle) was one of the successful parameter set and the other two had parameters that had little noise (high reliable/unreliable ratio and very few unreliable cells) or too much noise (low reliable/unreliable ratio and a more unreliable cells). Although the discrimination improvement seems similar, absolute discrimination was lower in the high reliable case and higher in the low reliable case.

Supplementary Tables

Row	A	B	N(D$_x$)	E(D$_x$)
1	R	R	$R - R \sim 0$	0
2	R	U	$R - U \gg 0$	0
3	R/U	0	$R \gg 0$ or $U \sim 0$	$\gg$ 0
4	U	R	$U - R \ll 0$	0
5	U	U	$U - U \sim 0$	0

Table A: Possible contributions by cell x towards discrimination. Columns 1 and 2 show x's response state (R–reliable, U–unreliable, or 0–silent) to odors A and B. Columns 3 & 4 show the discrimination contributions for normal - N(D$_x$) - and extended - E(D$_x$) - training. As we are interested in contributions that distinguish A from B, x's silent for A are ignored. For normal training, rows 2–4 generate high contributions, while rows 1 & 5 have low contributions. With extended training, only cells silent for B generate high contributions.

	APL gain	PNs	PN→KC	APL→KC	distance from observed
1	7.00	0.00	0.05	0.275	0.11
2	8.00	0.05	0.00	0.30	0.11
3	8.00	0.10	0.05	0.30	0.12
4	7.00	0.20	0.20	0.25	0.12
5	7.00	0.15	0.00	0.275	0.13
6	8.00	0.15	0.10	0.30	0.13
7	7.00	0.15	0.05	0.275	0.13
8	8.00	0.00	0.35	0.275	0.14
9	7.00	0.35	0.10	0.25	0.14
10	6.00	0.15	0.05	0.275	0.14
11	6.00	0.20	0.05	0.275	0.14
12	7.00	0.05	0.00	0.275	0.14
13	8.00	0.05	0.15	0.30	0.15
14	8.00	0.30	0.05	0.275	0.15
15	6.00	0.05	0.10	0.275	0.15
16	6.00	0.25	0.00	0.275	0.15
17	8.00	0.30	0.15	0.275	0.15
18	8.00	0.25	0.00	0.30	0.15
19	7.00	0.00	0.00	0.275	0.15
20	6.00	0.15	0.10	0.275	0.15
21	7.00	0.30	0.15	0.25	0.16
22	7.00	0.00	0.10	0.275	0.16
23	8.00	0.30	0.00	0.275	0.16
24	7.00	0.40	0.05	0.25	0.16
25	8.00	0.05	0.05	0.30	0.16
26	8.00	0.00	0.10	0.30	0.16
27	7.00	0.05	0.10	0.275	0.17
28	8.00	0.35	0.20	0.275	0.17
29	7.00	0.15	0.35	0.23	0.17
30	7.00	0.30	0.00	0.25	0.17
31	8.00	0.35	0.00	0.275	0.17
32	7.00	0.10	0.25	0.25	0.17
33	6.00	0.00	0.10	0.275	0.17
34	6.00	0.05	0.05	0.275	0.17
35	8.00	0.20	0.25	0.275	0.17
36	8.00	0.00	0.30	0.275	0.17
37	7.00	0.15	0.10	0.275	0.18
38	6.00	0.10	0.05	0.275	0.18
39	7.00	0.20	0.25	0.25	0.18
40	6.00	0.00	0.35	0.25	0.18

Table B: The top 40 parameter combinations (out of 44,217) of the noise exploration model that produced results most similar to the ones observed in Figure 1C,D. The first 4 columns are values of the parameters. Column 1: the APL gain that determines the WTA based sparse code, e.g., a value of 5 means that when there is no noise (deterministic system), the top 5% of cells are active. Column 2: PN noise describes the amount of noise at the PN level. Column 3: PN-KC: the amount of noise in the synapses between PN and KCs. Column 4: APL-KC: amount of noise in the feedback synapses from APL to KCs. In columns 2-4, noise is given as a fraction of the component's value, and for percentage terms, it must be multiplied by 100. Thus, in the first row APL→KC noise is 0.275 of the synaptic strength or 27.5% of it. Column 5: The result of the optimization constraint, shows how far the model's results are from observed results. The lower the value the better the fit. The table shows that all 40 parameter combinations contain noise in the APL→KC connections in the range 0.25-0.3. Correspondingly, PN and PN→KC noise range from 0 to 0.35, with quite a few entries being 0. Thus, noise in these two components is less important to producing the stochastic code than noise in APL→KC connections, which seem to be essential for producing observed results.

	Accuracy			AUC		
	Rel.	Unrel.	all	Rel.	Unrel.	all
lda (mouse)	0.60	0.20	0.40	0.80	0.59	0.64
lda (fly)	0.57	0.28	0.50	0.72	0.72	0.83
knn (mouse)	0.40	0.50	0.10	0.74	0.39	0.59
knn (fly)	0.64	0.36	0.21	0.71	0.36	0.65
svm (mouse)	0.55	0.20	0.40	0.88	0.70	0.84
svm (fly)	0.64	0.14	0.42	0.93	0.66	0.82

Table C: Results of the performance of 3 linear classifiers/decoders on the fly and mouse datasets: lda - linear discriminant analysis, knn - k nearest neighbor, svm - support vector machines. The decoders were trained for 3 types of cells - reliable (Rel.), unreliable (Unrel.), and all (all) cells - as described in Methods (Data Analysis section), and in this table we show the performance results in terms of accuracy (the fraction of the test set that was correctly predicted) and the averaged AUC measure. For all three decoders, it is easiest to distinguish between odor classes when only reliable cells are considered. Similarly, when all cells are considered, it is also more discerning than the unreliable class of cells, and except for the fly lda set, less slightly less discerning than reliable cells alone. Unreliable cells also have some amount of discriminatory power based on their non-negative accuracy scores, and high AUC scores. For all cases, the training set contained 80% of the odor trials, and the test set contained the other 20%.

Fly ID	exp. fit	a	b	#rel.trials	#unrel.trials	#rel.odors	#unrel.odors	# odors	# trials	#cells
main*	0.99	0.02	0.61	5.26	7.24	6.11	29.03	7.00	6.00	124.00
110107_1	0.95	0.06	0.79	1.40	3.65	1.64	10.05	8.00	3.00	107.00
110108	0.99	0.04	0.71	8.64	7.67	9.74	25.55	8.00	4.00	136.00
110109_1	1.00	0.04	0.70	3.99	6.66	4.56	21.62	8.00	4.00	137.00
110109_2	0.89	0.07	0.61	4.27	3.70	5.21	10.98	8.00	4.00	132.00
09042009	0.98	0.02	0.90	3.51	3.55	4.11	13.35	8.00	5.00	161.00
110106_4	0.99	0.03	0.75	11.80	7.30	12.64	23.04	6.00	4.00	149.00

Table D: The response characteristics (Fig. 1) for all the flies that were examined. The first 3 columns give the fit of the exponential and the two parameters a and b of the fit equation, $y = ae^{bx}$. These columns are followed by the mean number of reliable and unreliable cells per trial and odor; the numbers are percentages. The trials column gives the odor with the maximum number of trials. The double starred rows are ones where the total number of trials is lower than # trials * # odors. In order, they are 25, 22, and 35. As can be observed, the average trend is that the number of unreliable cells increases with total trials. The single starred fly (first row) is the one that was used for the figures in the main paper. The datasets analyzed here are available in the Zenodo repository whose link is listed in the Data Availability section.

Mouse ID	exp. fit	a	b	#rel.trials	#unrel.trials	#rel.odors	#unrel.odors	# odors	# trials	#cells
164*	0.99	1.10	0.49	3.40	8.66	4.42	43.09	10.00	8.00	285.00
163	0.98	1.09	0.42	1.30	7.04	1.67	38.65	10.00	8.00	318.00
7	0.98	1.72	0.36	1.62	9.66	2.31	49.12	10.00	8.00	147.00
8	0.96	2.34	0.42	2.44	6.60	3.14	35.54	10.00	8.00	121.00
9	0.98	1.77	0.40	2.39	10.30	3.16	51.10	10.00	8.00	209.00

Table E: The response characteristics (Fig. 1) for all the mice that were examined similar to Table D. The first 3 columns give the fit of the exponential and the two parameters a and b of the fit equation, $y = ae^{bx}$. These columns are followed by the mean number of reliable and unreliable cells per trial and odor; the numbers are percentages. Unlike the mouse, all mice experienced the same number of total trials, and as a result, they have similar numbers of average number of unreliable cells per odor. The starred mouse (first row) is the one that was used for the figures in the main paper. The datasets analyzed here are available in the Dandi repository, whose link is listed in the Data Availability section.

Supplementary Methods

Data Analysis: supplement

Analysis of imaging data to analyze effect of p-value

We re-analyzed the data to examine if changing the threshold of significance affected the results of the paper. Increasing the significance threshold would be akin to weeding out cells, whose responses are due to noise rather than being genuinely responsive, e.g., cells with high background means and low signal means. We analyzed three aspects of changing the p-value: how does it change the number of reliable and unreliable cells? What effect does it have on the improvement in discrimination ability with extended training? and, how does it change the ability to discriminate similar versus dissimilar odors?

We answer these three questions by breaking the effect of changing p-values into two regimes. In the first regime we consider the effect of lowering the p-value. In the paper the significance threshold was p-value=0.01, and we considered p-values 0.005, 0.001, 0.0005 (Fig I). Reducing p-values decreases the number of responsive cells per trial, and decreases both the reliable and unreliable cell populations proportionally (panels A,D in Fig I; flies and mice, respectively; points to the left of the red circle in all plots). This change, however, does not affect the result of the paper, which is that unreliable cells with extended training improve discrimination of similar odors (panels B,E in Fig I), where p-values 0.01 and lower have a similar effect. Finally, panels C,F in Fig I show that similar odor discrimination is worse than dissimilar odor discrimination with normal training, which is consistent with experimental results [2].

Next, we consider the second regime: higher p-values 0.05 and 0.1. Higher p-values increase both, the number of reliable cells and the number of unreliable cells (panels A,D in Fig I). However, p-value=0.05 follows the trend of the first regime, p-value=0.1 (rightmost point in all plots) does not. This is because at this low threshold nearly all cells respond, so the increase in the number of unreliable cells is less than that of reliable cells. This difference for p-value=0.1 also has an effect on the other two properties: discrimination of similar odors gets worse with extended training for flies (panels B,C in Fig I), and only marginally improves for mice, and similar odor discrimination becomes nearly as good as dissimilar odor discrimination for flies and becomes better than dissimilar odor discrimination in mice (panels C,F in Fig I). This is because previously unreliable (and decorrelated) cells (Fig. 4) become reliable, and contribute during normal training. Consequently, similar odor discrimination improves in comparison with other p-values. And, extended training has little effect on discriminatory ability. Importantly, experimental results show that similar odor discrimination is not better, suggesting that p-value=0.1 is unlikely to reflect real activity.

Thus, this analysis suggests that varying the significance threshold does not affect the result of the paper, and that extended training improves discrimination because of unreliable cell contributions.

Analysis of full range of cell reliabilities: Fig F

The goal of this figure was to examine the full range of response reliabilities of the population of cells. One issue with the data is that cell reliabilities can only take a a specific set of values, e.g., 1/6 to 6/6 for flies because of the constraint that we only have a dataset with 6 trials; it is not possible to observe a reliability of 1/9 or 1/20. The plots show that the underlying range of reliabilities is much bigger. In panels A,B in Fig F, we re-examined the fly and mouse data in the paper, by focusing on the first 3 trials and picking all cells with a reliability of 1/3. If cells were to have a fixed frequency, we would then observe the same reliability for trials 4–6 (or 4–8 in panel B and 4–16 in panel C). However, we observed a range of reliabilities from cells that responded in only trial to those that responded in all three. In panel C in Fig F we examined a fly dataset where the animal had been subject to 16 trials. Panels D–F in Fig F show the theoretical predictions for the datasets in (panels A–C). We illustrate how this was done by using panel D in Fig F. Here, each row reflects the theoretical prediction of the reliability composition of cells for trials 4–6. We generated the predictions by sampling from a binomial distribution with a size of 3 (which was changed to 5 for E and 13 for F) and probability of 1/3 (which was changed for the probability of other rows)

Modeling and Theory: supplement

Parameter Exploration: The parameters used for the different models in Figure 3 are as follows. For all the models, the number of PN types was 50, and the number of KCs was 150. The number of odors was 6, with 6 trials per odor. The APL inhibitory feedback gain was set so that only the top 6-11% of cells were active without noise present.

First, we describe how we tested both models by exploring their parameter space. With either model, we generated parameter sets where we systematically varied parameter values within prescribed ranges described below. For example, for PN noise from 0 - 50%, we varied noise in steps of 10%. For others like the APL parameter, which followed a Normal distribution, we varied noise in smaller steps of 2-4%, over a shorter range from 15 to 45%. We then generated parameter sets by taking all possible combinations of the 6 noise parameters. Additionally, we also varied a few non-noise parameters like APL inhibition gain or type of APL$\rightarrow$KC synapses. Notably, the APL-KC inhibition gain increased as noise reduced the

average number of active KCs after APL inhibition. We varied the APL gain to give us top % of KCs in the range of 6 to 11, in steps of 1. The other parameter that we varied was the type of synaptic connection as we already mentioned above. For the multi-synapse model, we tried three different distributions for synapse strengths: a Gamma distribution because of work in the mammalian olfactory cortex data in [3] and analyzed in [4], constant strength based on prevailing MB models, and uniform synapses which assumes strengths within a tight range of 20% of the maximum. We also examined the effect of the average number of synapses by varying them in the range of 2-40 synapses. We found that synapse numbers beyond 15 do not change the results (panel E in Fig J). The figures in the main paper of parameter explorations and supplement, unless otherwise stated use a synapse number of 15 or 20. Lastly, we tested each parameter set between 7-10 times, and took the result to be the average of these runs. Thus, if a parameter set were to give optimal results for one run but not the others, it would be weeded out.

In order to assess how well the model – with any particular combination of the 6 parameters – fits observed data, we exhaustively explored parameter space, while constraining the model with the 5 conditions observed with data (Fig.1): ratio of reliable/unreliable cells, number of reliable cells/trial, number of unreliable cells/trial, number of reliable cells across all trials, number of unreliable cells across all trials.

For each parameter set, we judged its fit to observed data in 2 steps, similar to an $l1 - norm$ minimization procedure [5]. First, the model had to produce results for each constraint that fell within mean $\pm$ sem range of observed results. Parameter combinations that did not satisfy this condition were discarded for the next step. To illustrate, consider Figure 3C. The y-axis measures the number of reliable/unreliable cells for each PN noise value with the line denoting the observed ratio (Fig. 1). The observed ratio is achieved at a noise value of 80%. On the other hand, even high amounts of noise in the KC$\rightarrow$APL synapses does not produce the desired result (Fig. 3D). We carried out a similar test for all 6 parameters. Second, for each of the constraints we took the absolute value of the difference between the model's and observed results normalized to observed results. For instance, for the constraint of reliable/unreliable cells, the ratio observed in experiments was 0.72, and what we observed with the parameter combination that was the global minimum for the single synapse model (APL gain = 7, glom. noise = 0, glom-kc noise = 5% or 0.05, APL-KC noise = 27.5% or 0.275) was 0.712. So, the result for this constraint was (0.72-0.712)/0.72 = 0.01. Normalization was necessary in order to ensure a comparison between different constraints. For instance, if we consider another parameter, like the number of unreliable cells across all trials, its value for observed data is around 30. Thus, it is an order of magnitude higher than 0.72, the reliable/unreliable ratio constraint, and normalization is necessary for comparison.

Parameter explorations for the Single synapse model: Table B

Figure 3 provided us with two characteristics of each individual parameter. First, each parameter had a monotonic effect on constraints (features in Fig. 1), i.e., increasing the parameter either increased or decreased these constraints. Second, it gave us the maximum value that yielded observed results. These two characteristics led us to using the following parameter range in our explorations: APL gain from 5 to 13 in steps of 1, noise in PNs from 0 to 80% of firing rates in steps of 5%, noise in glomerular$\rightarrow$KC connections from 0 to 80% in steps of 5%, and noise in APL$\rightarrow$KC connections from 0 to 40% in steps of 2.5%. Thus, in all, we explored 44,217 parameter combinations. And, we ran each parameter combination 7-10 times, and took the mean of the results.

We did not include noise in the connections from KCs to APL, because as we showed in Figure 3, even noise at the level of 400% does not produce observed results. This KC$\leftrightarrow$ APL connection has little effect on the stochastic code. We did not vary other parameters such PN firing rates or PN$\rightarrow$KC connection characteristics (no of claws etc.) as we used empirical values that were uncovered in [6]; [7] and described in [8].

Parameter explorations for the multi-synapse model

We did a similar analysis for the multi-synapse model. In this case, because of the increase in parameters, and the attendant combinatorial expansion of parameter search: e.g., with 5 parameters changing and one fixed if the number of parameter sets is 6,000, adding 3 points to the 6th parameter would increase the number of points to 18,000. So, we did the exploration in 3 stages. First, we used a coarse model with a larger range and big steps to fine-tune parameter space, and then a more fine-grained model with a smaller range and steps. We also did a combination for exploring parameter relationships like APL vs. KC noise, by using a combination, i.e., taking smaller steps in parameter space for these two parameters, while using a coarser model (larger steps) for other parameters. We give our fine-tune parameters below, and the variations for individual parameters used in analysis shown in figures after that.

List of parameters for the multi-synapse model:

1. PN noise: 0 - 50 %, steps of 10.
2. PN-KC noise: 0 - 50 %, steps of 10.
3. APL-KC noise: 0 - 50 %, steps of 10
4. KC noise: 10 - 50 %, steps of 10

5. APL noise: 20 - 50 %, steps of 2.5
6. APL gain: 6 - 11, steps of 1

Parameters used in figures:

1. Fig. 3E: PN, 0 - 40 %, steps of 5.
2. Fig. 3F: APL, 20 - 40 %, steps of 2.5.
3. Fig. 3G: KC, 0 - 50 %, steps of 10.
4. Fig. 3H: KC, 0 - 50 %, APL, 20 - 40 %, both steps of 10.
5. Panel A in Fig J: APL gain, 5 - 10, steps of 1
6. Panel B in Fig J: APL-KC, 0 - 50, steps of 10
7. Panel C in Fig J: PN-KC, 0 - 50 %, APL, 25 - 45 %, steps of 5 and 2.5.
8. Panel D in Fig J: Left: Gamma distribution, 6 APL-KC synapses; Uniform distribution, 20 synapses. APL mean: 0.42, and 0.32 respectively.
9. Panel E in Fig J: APL-KC synapses from 3 distributions: Γ(1.16,0.12), constant strength 0.4, uniform distribution (0.4,0.45).

For panel E in Fig J, there was no noise in the system. We recorded the KC neurons that fall in the top 15 %, and then recorded the numbers that were active after APL inhibition. We used a lower APL gain (i.e., with more top responding neurons) than our other model simulations,.

Fig G: modeling supporting the main results

In Figure 2, we showed the response sizes of all odor-KCs pairs from every trial. Here, in panel H in Figure G, we show the responses for one sample trial, again fit to a Gamma distribution with shape=1.12, and scale=0.32.

Panel J in Fig G shows the probability distribution function for 2 curves with Gamma Distributions, with shape = 0.22 (left) and 0.32 (right). The change in shape makes the distribution narrower from the left panel to the right panel. The narrower shape reduces the amount of noise needed to generate a stochastic code.

Fig G and Table B: exploration of noise in the single synapse model of the fly olfactory circuit

In panels E–G in Fig G, we mimicked realistic responses to odors in the absence of APL inhibition, i.e., the basal response without WTA, by first fitting data from [2] that was generously shared with us by Andrew Lin and Gero Meisenbock. We found that the response fit a Gamma distribution with shape = 4.12 & scale = 5.12 (shown in panel J), and panel E shows the response of a 100 cells drawn from this distribution and sorted by increasing order of response size. The next panel shows the response sizes, wherein the WTA inhibition is set to the top 10th percentile. Panel G shows the same responses, except this time the inhibitory feedback has multiplicative noise added to it with an amplitude of 20%.

Table B lists the model parameters that give the closest results to observed data in Figure 1. In order to determine the optimal parameters, we took four of the parameters of the circuit: the APL gain that determines the number of KCs that are active, noise in the firing rates of the PNs, noise in the connection synapses between PNs and KCs, and noise in the inhibitory feedback from APL to KCs.

The parameter combinations listed in Table B are the top 40 combinations arranged in increasing order of their distance – shown in column 5 – from observed results. The first combination is the global minimum. In all, out of 44,217 parameter combinations, 1290 yielded viable results, i.e., they satisfied each of the constraints. Amongst these, of the top 200 parameter combinations, 173 of them had APL-KC noise in the range of 20 - 30%. By contrast the range for PN and PN-KC noise went from 0 to 80%, suggesting that APL-KC noise was more significant and essential for producing a stochastic code. Additionally, in cases where there was no APl-KC noise, the amount of noise in the other two parameters jointly was in the range of 75 – 110%, showing that abnormally high amounts of noise is required in order to generate the stochastic code.

Figures 3, J, and K: The multi-synapse model

Here, we describe the modeling specific to the multi-synapse model, by describing the various plots in the Figures.

We also showed the effect of noise in various components of the circuit. The parameter distributions for APL noise was Normally distributed, while for PNs, PN→KCs, and KCs it was uniformly distributed, except at higher levels of noise, greater than 50 % for PNs and PN→KCs, and greater than 40 % for KC. the uniform distribution in these regimes suggests that the stochastic code is independent of specific values of PNs, PN→KC, and KC noise and depends on specific values of APL noise. Our results, however, highlight another facet of how multi-component noise interacts. The amount of APL noise needed is reduced when there is noise in KCs (Fig. 3H). It lowered the mean of the distribution from 0.32 to to 0.28. Similarly, the number of APL→KC synapses and the type of distribution also lowered the mean of the APL noise as observed in panel D in Fig J, where the mean shifted from 0.42 for the Gamma distribution with 6 synapses (on average) to 0.32 for the uniform distribution with 20 synapses. Increasing the number of synapses improves the channel capacity from APL to KCs, and thus improves the communication of APL signal that in this case includes noise, too.

The effect of connectivity at the APL→KC synapse is also illustrated in panel E in Fig J. Here, we experimented with the three distributions: Gamma distribution with shape = 1.15 and scale = 0.12, a uniform distribution with range of (0.4,0.45), and synapses all of the same strength. There was no noise in the system. We then varied the number of synapses as shown on the x-axis. For instance for the number 16, we generated three fly circuits with the three distributions. We then did a simulation with 6 odors, and measured the neurons that were in the top 15% without APL feedback and that were active after APL inhibition. We recorded the percentage of neurons that remained in the top set of responding neurons, and repeated this 10 times. The mean is plotted on the y-axis.

In Fig K, we show that the model results are independent of the number of KCs (size of the MB). To assess the effect of numbers, we tested for 5 properties of the stochastic code: the ratio of reliable/unreliable cells per odor, number of reliable and unreliable cells across all odors, improvement in useful discrimination (Fig. 6) with extended training for dissimilar and similar odors. In panel A, we plotted a comparison of observed results versus results from the model with 150, 250, 500, 1000, and 2000 KCs. The parameters here are: APL inhibition gain of 9, APL noise of 35 %, APL→KC, PN, and PN→KC noise of 10%.

In panels B–D of Fig K, we show that their response properties, similar to Fig 2, do not change with MB size. All cumulative plots are fits to Gamma distributions. Note that the distributions are different from the ones in Fig 2.

In panel E, we showcase three examples of the fly circuit. One that was successful at recapitulating the results of Panel A, shown with a black circle. The parameters are APL inhibition gain of 8, APL noise of 24 %, and 10% noise in the other parameters. The other two examples of non-successful models in that they failed to recapitulate all of the properties of the stochastic code. The noise parameters are very low noise < 10 %. The other example has a high level of noise, 50 % for APL and KC noise, as well as 20 % noise in PN and PN→KCs.

References

[1] Hallem EA, Carlson JR. Coding of odors by a receptor repertoire. Cell. 2006;125(1):143–160.

[2] Lin AC, Bygrave AM, de Calignon A, Lee T, Miesenbøck G. Sparse, decorrelated odor coding in the mushroom body enhances learned odor discrimination. Nature neuroscience. 2014;17(4):559–568.

[3] Schikorski T, Stevens CF. Quantitative fine-structural analysis of olfactory cortical synapses. Proc Natl Acad Sci U S A. 1999;96(7):4107–12.

[4] Srinivasan S, Stevens CF. The distributed circuit within the piriform cortex makes odor discrimination robust. Journal of Comparative Neurology. 2018;.

[5] Boyd S, Boyd SP, Vandenberghe L. Convex optimization. Cambridge university press; 2004.

[6] Bhandawat V, Olsen SR, Gouwens NW, Schlief ML, Wilson RI. Sensory processing in the Drosophila antennal lobe increases reliability and separability of ensemble odor representations. Nature neuroscience. 2007;10(11):1474–1482.

[7] Caron SJ, Ruta V, Abbott LF, Axel R. Random convergence of olfactory inputs in the Drosophila mushroom body. Nature. 2013;497(7447):113–7.

[8] Stevens CF. What the fly's nose tells the fly's brain. Proceedings of the National Academy of Sciences. 2015;112(30):9460–9465.
