# Supplementary material for: Effects of stochastic coding on olfactory discrimination in flies and mice
Source: PLoS Biol. 2023 Oct 31;21(10):e3002206. doi: 10.1371/journal.pbio.3002206 (PMC10618007; doi:10.1371/journal.pbio.3002206)
Supplement: Attachment — Submitted filename: reviewer_response.pdf [file pbio.3002206.s016.pdf]

Stochastic coding paper reviewer response

We thank both reviewers for their thoughtful and constructive comments. We have addressed all the concerns expressed in a point-by-point format below. In addressing the concerns, we added new results, which, in our opinion, have improved the quality of the paper. This has required a rewrite of significant portions of the modeling and methods sections. Additionally, we have made extensive changes to improve clarity and readability as suggested. In the revised manuscript, any new text is in red font along with previous text marked by a ~~strike-through~~ to simplify review. We have also included a version that does not have the changes highlighted in case that would be preferred. Our point-by-point responses follow.

Note: The main paper has been changed to accommodate the official PLOS format, which includes Materials and Methods in the main paper before results. So, the Methods figure is now Figure 1, as a result of which all the other figure numbers have increased by 1. So the old figure X is new figure X + 1. We have not changed the figure numbers in the reviewer comments, but, our references to paper figures take this change into account.

Reviewer 1

We thank the reviewer for their thoughtful and incisive comments, which have helped us significantly improve the paper. Below, we provide our responses to the reviewers' concerns, which are highlighted in *italics*.

1. *I find the overall framing of the manuscript to be somewhat unclear. The initial paragraphs of the introduction set up the paper as a test of the hypothesis that a sparse-coding-like scheme is implemented in the olfactory system, but the remainder of the paper lacks such a clear direction. At some points the reader is led to believe that this is a paper about possible computational roles for trial-to-trial variability, while at others the focus is again on a comparison to sparse coding. The current organization of the figures likely contributes to the lack of clarity; moving the discussion of different strategies for fine discrimination, and what is currently Figure 7, to the Introduction might help guide the reader.*

   We agree that this should have been made clearer. We set up the paper to challenge sparse coding on the basis that there also exists trial-to-trial variability, which is often ignored. The paper proceeded to demonstrate that such trial-to-trial variability exists in data and then proposed a computational role for it, within the context of sparse coding. Thus, our intention was to synthesize these two features of neural representations, which are often studied in isolation. To make this clearer, we added a new first paragraph that lays out our broader goal: There are two often-cited features of neural representations. The first is that only a small set of neurons fire in response to each stimulus (called sparse coding) that aids discrimination. The second is that a somewhat different set of neurons respond each time the same stimulus is presented (called trial-to-trial variability) that could hinder discrimination. Here, we seek to understand how these two features can work together to enable both coarse and fine odor discrimination.

   We decided to keep Paper Figure 8 at the end, since in this manuscript, we only explored one of the three possible mechanisms (cloud saturation), and the other two remain conceptual. Moreover, Paper Figure 8 assumes a mushroom-body-specific model (which would require additional text to introduce), and we did not provide experimental validation of this model. However, we now mention that this is the direction we're moving towards in the list of questions in the Introduction: ... Fourth, do cells with different levels of variability contribute differently to odor discrimination?

   We also rearranged sentences in the preceding paragraph, the fourth paragraph now, to motivate the questions better. We have included the following sentences. To explain cognitive processes downstream of peripheral sensory coding, neuroscientists must contend with a second issue that is closely tied to the problem of overestimating sparsity: variability in neural responses across trials, and the following sentence at the end, This variability (noise) and lower sparsity begs the question of how animals can produce robust decisions from a single exposure of a sensory stimulus. One can envision real world scenarios, e.g., sensing a predator, that might not afford the luxury of multiple trials for decision-making.

2. *Some discussion of the idea that the brain might perform sampling-based probabilistic inference (eg. work of Lengyel, Pouget) would be useful, particularly in how this idea might relate to the coding scheme proposed here. On first reading, these ideas appear compatible, and both ascribe a computational role to neural variability.*

   Thanks for this suggestion! We added the following to Discussion to mention the possible relationship between stochastic coding and sampling-based probabilistic inference: Trial-to-trial variability has also been viewed as the brain sampling from a probability distribution of possible outcomes (Pouget et al., 2013). These models require that the animal first learn a generative model of the environment (including a likelihood function and a prior). Stochastic coding may provide a simple implementation of this scheme, where the reliability of a cell can be viewed as the probability that the features encoded by that cell are present in the odor.

3. *The discussion of the ubiquity of trial-to-trial variability in neural systems—as well as its possible benefits—is a bit vague; it should be revised to specifically address the question of variability in early sensory coding. In doing so, it is important that the authors distinguish between the different timescales over which responses may vary, and the circuit mechanisms to which variability may be attributed. In that vein, it might be useful to cite a review on longer-timescale representational drift, such as Rule et al 2019 or Masset et al 2022. It is also not sufficiently clear whether the phenomenon of stochastic resonance is directly relevant.*

   The MB/PCx are 3rd order neurons, which may be viewed as "early", but there is extensive data to suggest that they play important roles in "higher" order processes, including learning and memory (Haberly, 2001; Johnston et al., 2005). We also cited references that found variability in both earlier and later layers because in our model, we introduced variability in different layers along the olfactory tract. To make this clearer, we added: ... variability has been observed in both early sensory coding areas, as well as deeper layers involved more directly in learning and memory.

   Thanks for mentioning representation drift. As suggested, we added: Our focus here was on neural response variability over the time-scale of seconds (i.e., repeated trials in succession). Representation drift (Rule et al., 2019) is another form of variability where single neuron responses to a stimulus continuously change over time; however, this type of variability typically occurs over much longer time-scales (e.g., days to weeks (Schoonover et al., 2021)).

Finally, regarding stochastic resonance, we thank the reviewer for pointing it out. The connections were unclear in the previous version. We shortened the stochastic resonance section and drew a better analogy between it and variability. Stochastic resonance, through noise increases the range of sensitivity to sensory signals. We hope the following text makes the analogy better: The WTA mechanism is analogous in creating a central-SR process as it reduces the WTA inhibition based threshold of KC activation through noise, and allows less reliable cells to play a role in discrimination. This noise enable the animal to discriminate a range of odors from dissimilar to similar. We hope this makes it clearer.

4. *The current title could lead the reader to believe that this is the first paper to propose a concrete computational role for noise in neural systems. This is clearly not the case, and the authors might consider adopting a more specific, precise title.*

   We changed the title to better reflect our three contributions: showing that odor coding is stochastic; that this feature is conserved in flies and mice; and that this feature could affect odor discrimination. The new title is: "Stochastic coding: a conserved feature of odor representations and its implications for odor discrimination"

5. *The discussion around Figure 6 seems needlessly confusing. It would be more useful to describe the model and show the result in Figure 6 without the example numbers or Table 1.*

   As suggested, we removed all parentheticals with the example numbers, and we moved Table 1 to the Supplement.

6. *The conceptual discussion of models for fine discrimination in Figure 7 seems overly simplistic, as it treats the code as roughly binary and does not address the structure of noise. In particular, this is important since noise scaling or addition could blur any of the distinctions among representations of different odors.*

   We agree; Paper Figure 8 was only meant to demonstrate simple concepts, and many details need to be worked out to properly analyze them. We think this is in line with many other papers that end papers with a schematic model showing the main "take-aways" from their study. We added the following to now mention these caveats: Importantly, these are only schematics of plausible models, intentionally simplifying certain details such as firing rates of responding neurons, noise structure, and other possible read-out mechanisms.

   In flies, KC responses do seem to be nearly binary — firing either zero or just a few spikes per odor [Stopfer et al., 2003; Wang et al., 2004; Turner et al., 2008]. In mice, binary responses has also been suggested in the PCx [Bolding and Franks, 2017], where odor classification done with spikes or binarized responses give similar results.

7. *It is vital that the authors provide more justification for the criteria used to classify cells as "significantly responsive" and "reliable." Indeed, the authors themselves discuss the fact this distinction is "somewhat arbitrary." Whether these choices are merely useful for exposition or profoundly affect the qualitative results should be made clearer. One possibility would be to show how sensitive subsequent analyses are to the thresholds chosen (to define responsive vs not responsive). For a large part of the paper, the definition of reliability seems to be based on binary "respond" or "not" - rather than differences in firing rates.*

   Related: the view of defining unreliable cells in a SINGLE trial is a bit confusing because you can only know whether a cell is "unreliable" or not from multiple trials.

   The reviewer brings up three very relevant points: 1) How do we decide whether a cell is responsive? 2) What is the criterion for deciding that cells are reliable or not? and 3) Does varying the threshold qualitatively affects our results?

   For the first question, for both flies and mice, we decided whether a cell is responsive using criteria established previously in our labs (Turner et al., 2008; Campbell et al., 2013; Roland et al., 2017) and others (Woods et al., 2020; Stettler & Axel, 2009). Briefly, for each cell, we calculated its mean background response before odor onset, and selected a cell as being responsive if the mean for a set time after odor onset was above a threshold number of standard deviations (SDs) over the background mean. The threshold number is determined by the p-value, which is 0.01 in the paper corresponding to 2.33 SDs above the background mean.

   For the second question, to decide whether a cell is reliable, we used previously established criteria (Campbell et al., 2013; Turner et al., 2008), wherein if a cell responded significantly in more half the trials, it was reliable. But, as we acknowledge earlier in the text now (first section of results), the criterion for dividing reliable and unreliable is somewhat arbitrary, and we had used it purely for the purpose of exposition. Indeed, as we show in Paper Figs. 3–6, we analyze the cells on the basis of their graded reliability, i.e., the number of trials in which they respond, rather than if they fall into (binary) reliable or unreliable classes. To make this clearer, we have also made changes throughout the text to emphasize the graded nature (continuum) of reliability and attendant properties in cells. We find that the more reliable the cell, the less it contributes to discriminating similar odors. We agree with the reviewer's point, and have included additional data in the revised manuscript that shows the number of cells at each reliability level, their discriminability, and how specific (biased) a cell is towards an odor depends on reliability in a continuous manner. These figures are now part of Paper Figures S4D–G and 7E,F. Also, the following text was added to the first paragraph of the first section of results: This division into two classes is used to ease exposition and to serve as a comparison to earlier work

(Turner et al., 2008; Campbell et al., 2013); in the next section we show that these cells actually fall along a reliability continuum.

For the third question, we revisited the original datasets analyzed in the paper, and examined how changing the significance threshold (mentioned above) for a KC affects two properties: the ability to discriminate odors, and if the ratio of reliable to unreliable cells changes (Fig. 1). We considered 5 thresholds: 0.05, 0.01, 0.005, 0.001, and 0.0005. The plots in the main paper use a significance of 0.01, which corresponds to responses whose mean in the odor onset period is 2.33 standard deviations above the background mean. We find that increasing the threshold reduces the number of responsive cells, which reduces the number of cells in each of the reliable categories; however, the relationship between significance and ratio as shown in Fig. 1 (below) remains linear; i.e., the ratio remains the same. Importantly, as shown in Figs. 1C,D, discrimination still improves at higher thresholds (all ratios > 1). Parts of Fig. 1 and the explanation given here is part of section on “Analysis of imaging data” in the supplement and Fig. S9.

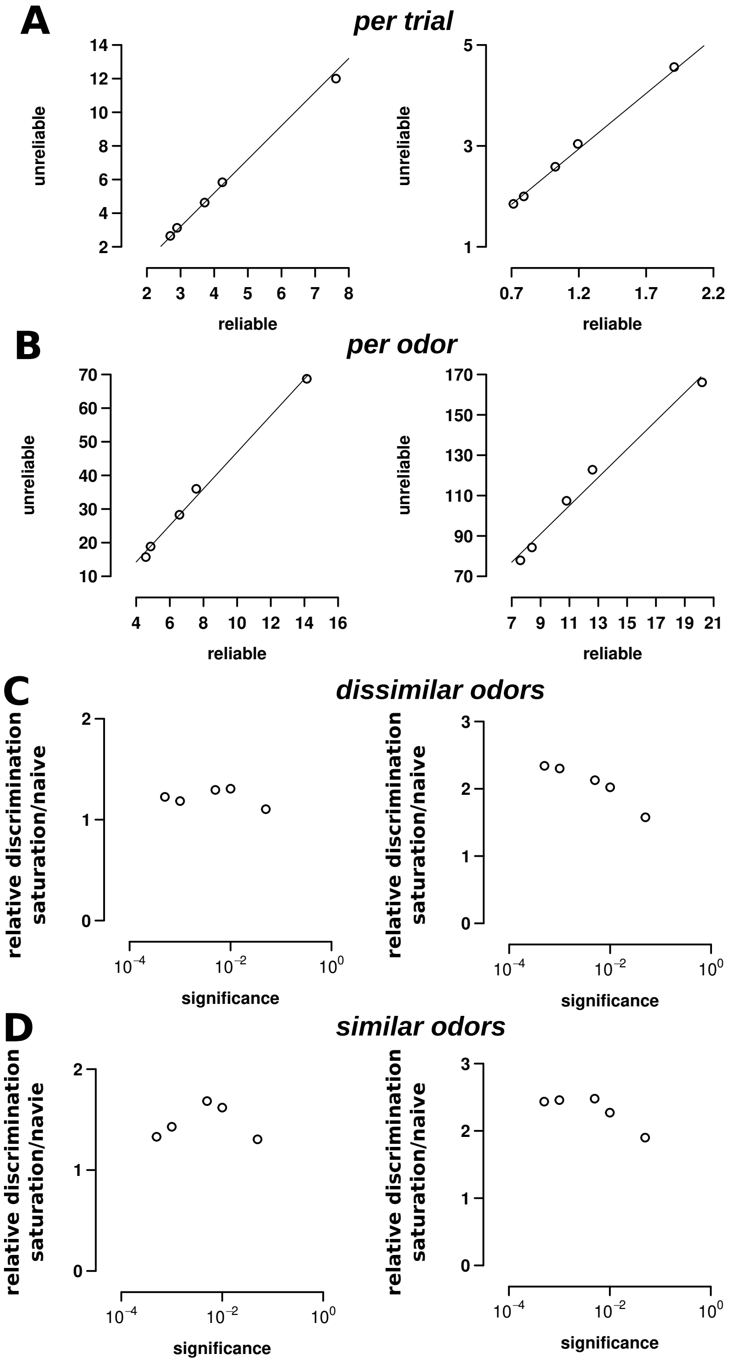

Figure 1: **Effect of changing thresholds on cell reliabilities and discrimination**. For A–D, fly data is on the left, and mouse data is on the right. (A) Changes in the number of responsive reliable vs. unreliable cells per trial with change in α, the significance level; α increases from 0.0005 on the left to 0.05 on the right; $\alpha = 0.01$ in the main figures. As α increases, more cells respond, and the increase in reliable and unreliable cells is proportional. (B) Changes in reliable vs. unreliable cells per odor. Again, the increase in number of cells with α is proportional. (C, D) Relative discrimination contributions of unreliable and reliable cells with extended training vs. with normal training plotted against significance, for dissimilar odors. Changing α does not significantly change discrimination ability. For both C and D changing α also does not change the relative discrimination ability between dissimilar and similar odors.

About defining unreliability based on single trials, we apologize if the text was misleading. The property of reliability is defined based on responses on all trials. Part of the confusion might come from referring to unreliable cells per trial. In this case, we split responding cells within a trial into those that are reliable or unreliable across all trials. We clarify this in the text, by adding the following sentence to the first paragraph of the first section of results: Note that the reliability property of a cell is determined by its responses over all trials. We also changed the previous sentence where we said cell 1 with a reliability of 3 out of 4 trials instead of just cell 1 with a reliability of 3.

8. *The motivation for using multiple classifier types in the decoding analysis is unclear. Why not just use a linear decoder, for which one would have a clear idea of what features of the response are relevant? Also, the method used to isolate reliable and unreliable cells for the discrimination analysis seems peculiar. Instead of setting the responses of cells in*

some sub-population to zero and then performing the decoding analysis, why not just exclude that sub- population? Also, why is the cross-validated accuracy not used?

Having multiple classifier results, when all of them show similar qualitative results, may indeed be excessive. Accordingly, we have taken out kNN and SVM, and moved them to the supplement. Two of the three classifiers we used (LDA and SVM) are linear, and kNN is non-parametric. We used all three because their underlying classification strategy is different — LDA is Bayesian, kNN is distance-based, and SVM seeks separating hyperplanes — and finding similar results for all three lends more strength to the conclusion we can make.

Re: zeroing-out sub-populations — The problem with taking out sub-populations is that the number of reliable or unreliable cells varies for each odor. Thus, taking them out gives you input vectors of varying lengths per stimuli, which becomes a problem in typical classification setups. We felt that turning these cells to 0 across a sub-population would render their contributions null, while keeping all vector lengths the same. For instance, kNN distances will be the same whether the sub-population is removed or zero'd out.

Re: using two different metrics — In the first part of the results (Fig. 5E), we tested the ability to identify odors, which the reliable cells were important for. With the second part of the results (Fig. 5F), we used AUC because we tested pairwise discrimination of all odors. For this task, AUC was a better metric, as it tells you how good the algorithm is at separating odors A and B. As shown in Fig. 3D, discriminating similar odor pairs ones had a lower AUC than discriminating disssimilar odors, on average. The procedure for the second part of the results involved training with a training set, and then testing with a held-out test set of odor trials. The only difference was that we used the AUC metric to show the probability that the algorithm would make a mistake. We felt this was a truer representation of the pairwise discrimination experiments that are usually carried out and the discrimination scenarios outlined in this paper (Paper Figs. 7 and 8).

To clarify, we did use cross-validation accuracy on both sets of results. In both cases, the data was broken into two sets for training and testing. In the first case – examining how well the algorithms performed odor categorization – the training set size was 80%. In the second case – examining how well the algorithms could discriminate between odor-pairs – the training set size was again 80%, and the test set was used for predicting the odor and obtaining a matrix of odor-predictions $Pred.mat$. The rows were the presented odors, and the columns were the set of odor classes. Each entry is the probability of the odor i being classified as odor j. The AUC score for odor i and j written as $AUC(i|j)$: the probability that a random trial of odor j has a lower probability of being classified as being in class i than in class j. The $AUC(i|j)$ calculation is based on the following equation.

$$AUC(i|j) = \frac{S_i - n_i(n_i + 1)/2}{n_i n_j} \tag{1}$$

The entries in this equation will become clearer if we explain the algorithm used. We take all the test odors from the odor classes i and j that were presented, and calculate the vectors f_i and f_j. f_i contains the entries (from $Pred.mat$) for all the test odors from class i and their probability of being classified as odor i. Similarly, f_j contains the test odors from class j and their probability of being classified as odor i. n_i and n_j are the number of entries in f_i and f_j. We rank order the entries of f_i and f_j, and then S_i is sum of all the ranks for the entries from f_i. We repeated this procedure for $AUC(j|i)$ and took the average so $A(i,j) = (AUC(i|j) + AUC(j|i))/2$. The AUC score for the dataset is the average of all odor pairs (i,j).

We have now included this explanation in the methods describing how we calculated the AUC score. The accuracy and test scores are given in Table S3.

We added the following to the main text: Given an odor-pair A and B, the AUC quantifies how well the pair can be discriminated. A score of 1 indicates high discriminability while 0 indicates a high likelihood of mistaking one for the other (see Methods for details).

9. *Figure 4C. This seems like a really bad comparison: if you look at the y axis, the range of values for similarities is very small and around 0. This is likely simply a consequence of unreliable and weak responses - so saying there is a lack of correlation is not justified. It would be more interesting and significant if the range in the y axis had been large and then there is no correlation.*

   We agree. We decided to keep the panel in, so as to not "hide" anything, but we changed the text to read: When splitting KCs into two classes, we found that reliable KCs well preserved the similarity of OSN representations and had a large slope ($r = 0.80$, slope=0.88; Paper Fig. 5B) compared to unreliable KCs, whose range of similarity values was much smaller (Paper Fig. 5C), likely due to infrequent and weak responses.

10. *The statement that "a winner-take-all mechanism is required for generating stochastic codes" is too strong; the results here merely show that such a mechanism is sufficient within the given model, while a subset of others is not.*

We softened this sentence to say that the WTA mechanism is the “best candidate” for generating stochasic codes. We also added other possibilities: Moreover, our results that the APL loop is the best candidate to generate KC response variability may need to be revisited if the Drosophila connectome reveals other connections, such as KC-to-KC feedback loops, or if the model is applied to the mouse olfactory system, where there are a population of inhibitory neurons that play the role of APL.

In addition, we updated the model to take into account the latest constraints from the Drosophila connectome and related studies (Abdelrahman et al., 2021; Prisco et al., 2021; Baltruschat et al., 2021). The changes specifically had to do with the connections from KCs to APL and back. Studies had shown that the synapses are individualized, while in our previous model, synapses from APL back to every KC had the same strength, and synapse noise was correlated. Thus, with global inhibition and no noise, we could precisely predict how many of the top N% of cells would be active. The updated model has different feedback strengths/sizes for each APL→KC synapse. The updated model also included findings from (Prisco et al., 2021) that the number of synapses from KC→APL were correlated with the number of synapses from APL→KC, for each KC. Finally, we varied the number of APL→KC feedback synapses in the range of 5–20 per KC, following (Li et al., 2020) who showed that this number is around 15 on average.

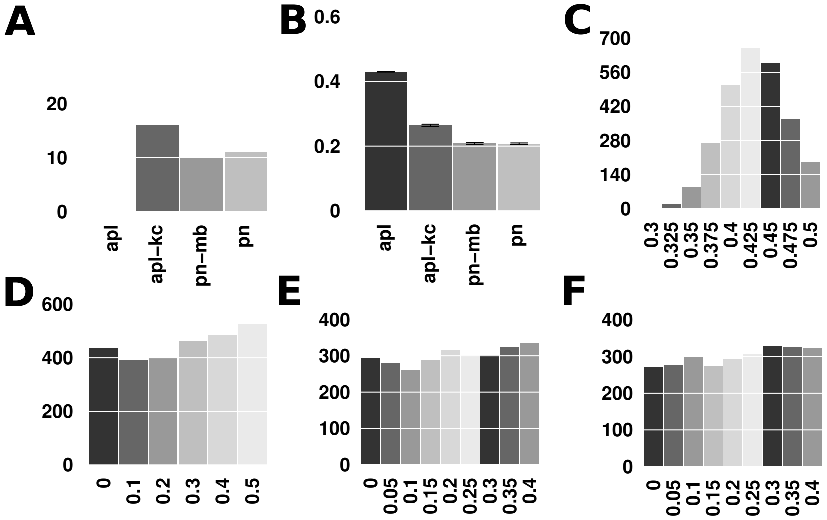

Exporation of the parameter space of the model. There are 4 parameters, whose noise levels were modulated over a range from 0 to 50%, and a point in parameter space correponds to one combination of these parameter noise values. The parameters were APL, APL-KC, PNs, and PN-MB. In addition, each combination was applied to models where the APL cut off all firing except the top 5-12%. A point in parameter space was considered successful if for those parameter values we were able to recapitulate the statistics shown in Figure 1. In total, we systematically examined 91,854 points. Of these 2,700 points recpaitulated observed results. The figures show the analysis results. (A) Percentage of parameters for which the parameter was not needed. The plot show that the APL was neccesary for observing experimental results. (B) The average values of the noise parameters. (C-F) The distribution of the noise values for the four parameters: (C) APL, (D) APL-KC, (E) PN-MB, and (F) PNs. The distributions for D-F are uniform, while that for C is normal (mean: 0.41, sd 0.04), highlighting the importance of APL noise. It shows that different levels of noise in the other three parameters produce observed results, while only specific values of APL noise produce observed results. Thus, the location of parameter space where the model produces realistic results is governed by APL values. In other words, despite it being 4 dimensional, actual parameter space is one-dimensional.

Figure 2: **Exploration of the parameter space for the updated model**.

With this modification, we found (Fig. 2, above) that the primary noise source that produced the observed results was noise in APL firing rates, as opposed to noise in APL→KC synapses, as we originally reported. This difference occurs for the same reason that PN→KC noise has little effect on KC variability over low-to-medium noise ranges: noise in PN→KC synapses gets averaged over multiple synapses as the source of the noise at each synapse is independent of the others. Similarly, noise in APL→KC synapses gets averaged. But, the averaging also means that the APL signal is reliably conveyed to KCs, and this extends to noise in APL. Which means that the top N% of KCs that are active varies per trial leading to reliable and unreliable cells, and the observed results.

Additionally, we report two other results. First, we did an extensive search through parameter space where we varied the APL-based KC threshold, noise in PNs, PN-KCs, APL, KC-APL, and APL-KCs (Fig. 2). In this parameter space, the observed results were not possible to recapitulate when there was no noise in APL. Further, the noise parameters for the other parameters varied uniformly over a large range (Fig. 2D-F), whereas it was a normal distribution for APL noise (Fig. 2C), which suggests that it is APL noise that most likely generates the observed results, though the amount of APL noise required is related to noise in these other parameters. These results are described in greater detail in the amended paper (Figs. 3,S11,S12) and methods (Modeling and theory) and also in points addressing similar concerns by the second reviewer.

11. *This is necessarily a model- and search space- dependent statement. As far as I can tell, the role of the inhibitory feedback synapse reliability is experimented only numerically - no analytical or conceptual reason given for why that synapse is the one. Also, could it be due to the assumptions of particular connectivity? For example, how will this WTA mechanism work when the feedback signal comes from a population of inhibitory neurons, rather than a single (or a few) APL-like cells? Does stochasticity across different inhibitory neurons need to be coordinated?*

    Re: conceptual reason — We apologize if the conceptual finding did not come through. With the revised results, we are making the following statement regarding the noise. Variability in KC responses is a result of (1) a mechanism/process that involves a feedback loop, and (2) this feedback loop has to be present in the layer or circuit that contains variably responding cells. In other words, noise in upstream layers like the nose or the lobe/bulb cannot, by themselves, induce variability in the subsequent layer because of the averaging of noise at the synapses of the KC dendrites. We added the following text to the paper: Conceptually, the model shows the limitation of noise sources in upstream stages of a circuit being able to produce variable responses in downstream targets. As we show, as each KC receives input from multiple PNs, there is an averaging out of noise from each of these sources. The feedback noise from APL, however, comes from a single source and cannot be averaged out.

    Re: assumptions of particular connectivity — For the WTA mechanism, in the updated model, we tested a few distributions for APL feedback to KCs. A gamma distribution (similar to mammals), a uniform distribution, and a constant distribution, which is the assumption that currently made in the fly community (that synaptic strength is equivalent to the number of synapses). We also tested the effect of the number of synapses on feedback (Fig. 3, below). The distribution chosen, i.e., the type of connectivity chosen, does have an effect on the amount of noise that produces observed results. Qualitatively the result still remains the same, i.e., the APL feedback loop remains the best candidate for variability. The modeling, however, does show that the APL network with the current assumptions of around 15 feedback synapses of similar strength from APL to KCs gives the results that best fit the data.

    Re: population of inhibitory neurons — The case of the population of inhibitory neurons is akin to the Piriform cortex. We did not tackle that case because the network structure is much less known. Among the major unknowns are the number of synapses between inhibitory and excitatory neurons, and even the breakup of different kinds of inhibitory neurons and their connectivity. For instance, it is well documented that some inhibitory neurons inhibit other inhibitory neurons (Canto-Bustos et al., 2022). Given the number of unknowns, we felt it was better to make headway with the simpler fly network. We should note that the fly model used parameters from published studies detailing the fly olfactory circuit and analysis of the published data (Caron et al., 2013; Gruntman & Turner, 2013; Stevens, 2015; Prisco et al., 2021; Baltruschat et al., 2021; Li et al., 2020; Amin et al., 2020). For instance, the number of connections from each PN to a KC, the strength of these synapses, the correlation in the number of synapses from API-KC and back from KC-APL. For parameters that are unknown, like synaptic distributions for instance, we tried various possible scenarios and over a range, too, to discover plausible parameter sets (as mentioned in previous paragraph: Fig. 3A-C). So, the model is constrained by data in the literature to the best of our ability.

    We now mention this caveat, as well: A similar WTA mechanism exists in PCx, albeit with multiple inhibitory neurons. Given the number of unknowns such as the number of inhibitory synapses, their distribution, and the number of inhibitory neurons feeding back onto one excitatory neuron, it is difficult at this stage to make a realistic model to assess the PCx WTA's contributions to variability.

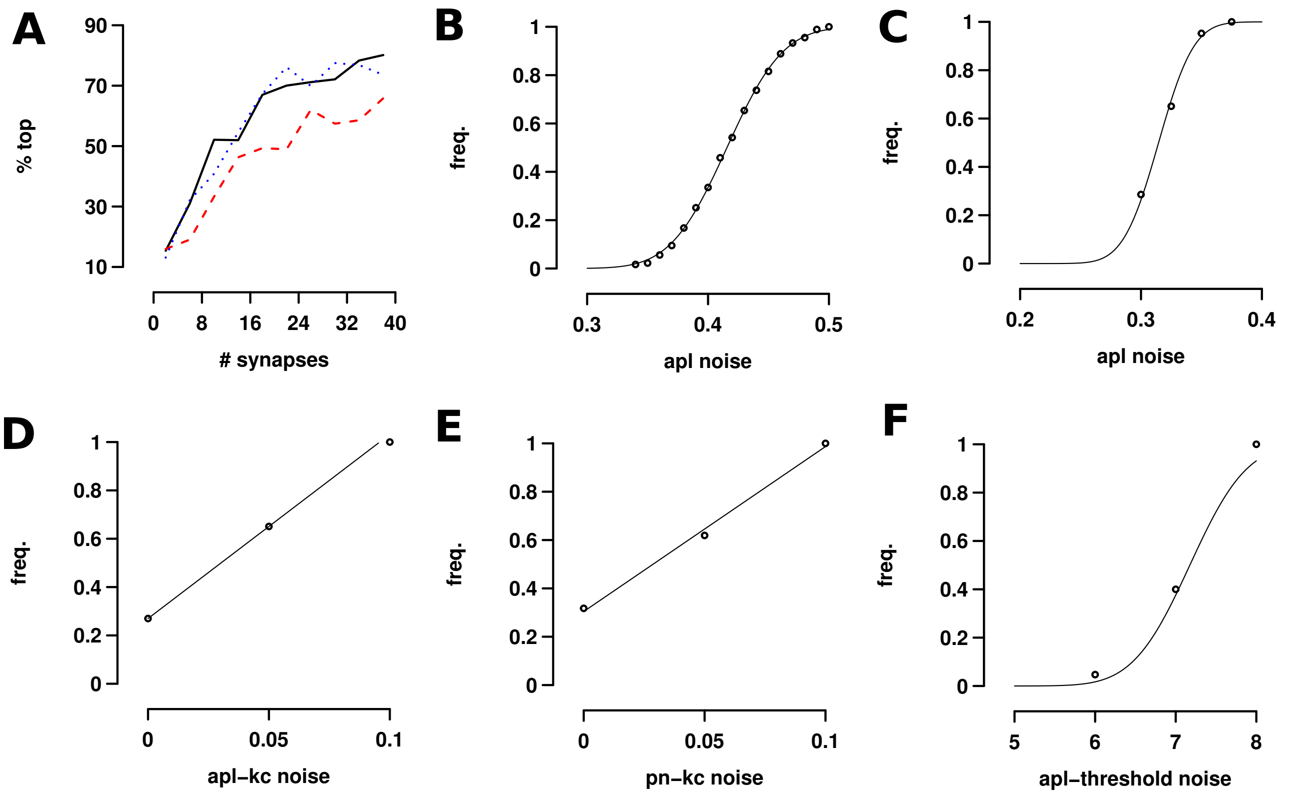

Effect of the synaptic distribution and other parameters on model performance. (A) Changing the distributions of the APL-KC synapses changes the composition of the top 15 % of the neurons. Here the apl-cutoff was fixed so that, on average, the top 15 % of cells fire. Each line is a different distribution: black: all synapses are the same strength, blue dotted: synapses are generated from a tight (small range) uniform distribution, red dotted: neurons are generated from a Gamma distribution. On the x-axis, you have the mean number of synapses for each APL-KC connection. The number for each APL-KC was generated from a Poisson distribution with this mean. The top % indicates the percentage of cells that remain as top responding neurons. This percentage increases as the number of synapses per APL-KC connection is increased and is also higher for the constant and uniform distributions. (B-C) the amount of noise needed in APL to generate successful results is reduced when we move from a Gamma distribution with 6 synapses (B) to a uniform distribution with 20 synapses (C). More synapses means less noise in the the APL inhibition signal to each KC, i.e., to the right of (A). (D-E) cumulative frequency histograms of noise in APL-KC and PN-KC connections. Both distributions are excellent fits for a uniform distribution indicating that noise in these parameters does not affect the performance of the model much. (F) APL threshold parameter thresholds are normally distributed.

Figure 3: **More detailed exploration of noise in APL and the synapse distributions in APL→KC synapses**.

12. *The quality and clarity of the figures could be improved, as they are often difficult to understand without careful reading of the caption.*

    We expanded on the labels for every figure panel to make their quantity understandable without reading the caption. A summary of the changes are below:

    Fig 2B: changed R to "Reliable" and U to "Unreliable" in 1B
    Fig 2E: mean response (dF/F) → mean KC response (dF/F)
    Fig 2H: mean response (dF/F) → mean PCx response (dF/F)
    Fig 3: moved blue "fly" text to top of row; same with blue "mouse" text
    Fig 3B: reliable → KC reliability
    Fig 3C: response amplitude → KC response amplitude
    Fig 3B–G: cum. freq. → cumulative frequency of cells
    Fig 3E: reliable → PCx reliability
    Fig 3F: response amplitude → PCx response amplitude
    Fig 4B: odor → glomeruli response
    Fig 4B: connection matrix → glomeruli-to-KC connection matrix
    Fig 4B: MB response → KC responses
    Fig 4: rel. → reliable
    Fig 4: unrel. → unreliable
    Fig 4C: annotate the dotted line in the figure
    Fig 5A: KC similarity score → Similarity score (All KCs)
    Fig 5B: reliable KCs → Similarity score (Reliable KCs only)

Fig 5C: unreliable KCs → Similarity score (Unreliable KCs only)
Fig 6E,F: UR → U
Fig 6A: cosine similarity → KC response similarity
Fig 6C: cosine similarity → PCx response similarity
Fig 6A,C: added figure legend for top circles/bottom circles = similar/dissimilar
Fig 7: spelled-out U, R, N, and E

13. Results of Figure 1E and H are entirely expected, right? That is, only cells that are weakly responding are likely to be unreliable (a simple consequence of peri-threshold variability?)

    Statistically, this is true as the reviewer notes from Paper Figures 2E and H. But, in our analysis, we did find a few cells that did not respect this guideline. Consider Paper Figures 6B and D. While most of the bottom 25% (by response magnitude) of cells are from lowest 2 reliability classes, the top 25% of cells were distributed across all reliability classes, suggesting that some low reliability cells neverthless have a high response. Based on our model, this could be the result of the odd high noise event.

14. *Figure 2: why is overlap calculated this way, rather than at a population level? something to do with number of simultaneously-recorded neurons? That brings up the question: how many neurons were simultaneously recorded, and are trial identities the same for all neurons (ie, simultaneous vs pseudo timed)?*

    We think the reviewer is questioning why overlap is computed at the cell level. The writing might not have been clear, but overlap data shown in the figure is the population overlap. Even though it is calculated at the single cell level, the results are summed over the entire population.

    Re: how many neurons etc., 124-150 neurons for flies, and 150-288 neurons for mice. Yes, the trial identities are the same.

15. Figure 2B/E: how will these curves change when the number of trials used to calculate reliability increases?

    Good question. As you increase the number of trials, the cells with responses in the fewest trials (1, 2, etc.) will constitute the most populous reliability class, but, note that as a percentage their number will be lower, as will be the number in each reliability class. So, we expect the cumulative curve to look similar, except now all the points will be more closely spaced. So, the difference in the number of cells with reliabilities x and $x+1$ will grow smaller. For instance, if we can extrapolate between mice and flies, consider Fig. 3B,E. The distributions are almost identical but the spacing between points is smaller.

16. *Figure 3AB: I find the fully linear model to be strange if you don't include a threshold nonlinearity, which is the essence of neural function.*

    This is correct. Normally, a threshold non-linearity is needed. There are two events happening at the KC, which have been modeled as a two–step process in this paper. First is the PN inputs that a KC gets. The second is the inhibition from APL, which suppresses a majority of the population, and acts as a natural thresholding mechanism, and the reason why we did not include a threshold for the KCs. But, as the reviewer correctly points out, thresholding is an essential part of the process. When we included a threshold, it did not change the results observed, so to ease computation, we left it out. This explanation has now been added to the Methods.

17. *The color bar in Figure 3H seems highly skewed, it looks nearly uniform.* This might have been an inkscape issue, but is now not part of the main figure.

18. *In the discussion of trial-to-trial variability as a driver of exploration, it would be useful to cite a more recent review on song learning, e.g., Chen & Goldberg, Current Opinion in Neurobiology, 2020. This is relevant only if the authors retain some discussion of the role of variability in behavioral learning.*

    We thank the reviewer for the reference. We added it to the Discussion.

19. *On page 7, the authors incorrectly characterize the Gamma distribution as the maximum entropy distribution with constrained mean E(X) and log-mean log E(X). The correct statement is that the mean E(X) and mean-log E log(X) are constrained.*

    Thanks for pointing that out. Now, fixed. Modified the maximum entropy paragraph of Sec. 2

20. *Figure 5E: I find the results counterintuitive - the sum of everything should always do better than a subset!*

    The reviewer raises a good point. We think that the reliable cells are intrinsic to an odor, while unreliable cells are not. Because of this, the addition of unreliable cells reduces accuracy. While an ideal classifier would ignore such noise, in practice, this does not always happen. For instance, consider a distance metric based algorithm like kNN. Here, the addition of unreliable cells increases the cloud of the region with which an odor resides with fewer odor-specific cells, and it decreases the distance between odor clouds.

We should stress, however, that unreliable cells are not random. If cells with a reliability of 6 for flies and 8 for mice have the highest bias (towards some individual odor), then cells with reliability of 1 have the least bias, though not completely random. We did an analysis by comparing (dividing) the overlap between cells from a reliability class versus cells chosen randomly. If the score is 1, it is the same as choosing cells randomly. As shown in Fig. 4, bias increases from the lowest reliability class to the highest, and even though cells with reliability 1 have a bias close to 1, it is still greater than 1, suggesting the presence of some bias.

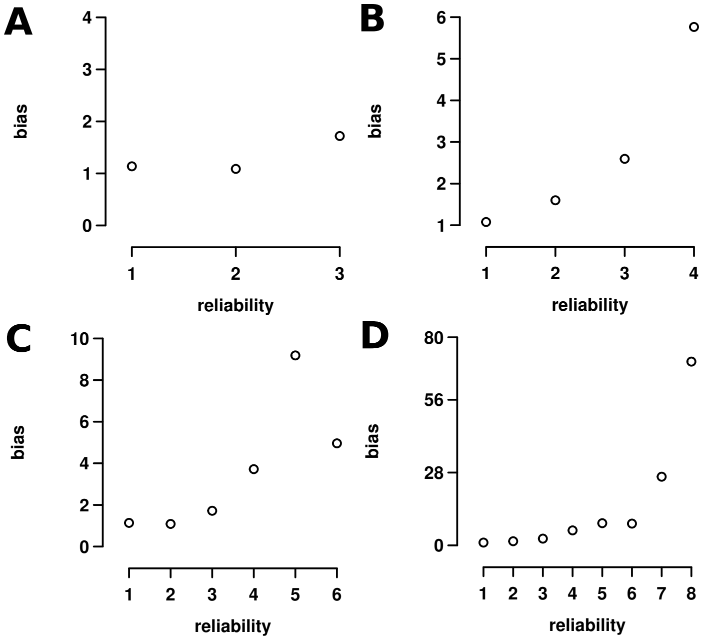

Bias or non-randomness of cells in each reliability class based on their overlap, in flies (A, C) and mice (B, D). (A-B) The bias for unreliable cell classes. Each point represents the average bias of cells belonging to a specific reliability class across all odor pairs. Bias is calculated as the measured overlap normalized to overlap o cells in that particular group if chosen at random. For instance for odors X and Y, the measured overlap is the number of cells that respond to both odors. The overlap for randomly selected cells is calculated theoretically. It is the expected value of overlap for all cells, where the probability of overlap of a single cell is the probability of the cell having a response to both odors. (C,D) Bias in cell overlap for all reliability classes.

Figure 4: **The selectivity (bias) of a cell for an odor increases with reliability**.

21. *"This measure is also the reliability measure of the cell used in previous figures (Figs. 1,2)." why add a different name for the same measure?*

Thanks for catching this. We agree and removed the old name for the measure. The text now reads: We developed a third property, which we call *overlap*, to capture the degree of similarity in cells' responses to pairs of odors. For each cell, we computed the probability that the cell will respond to both odors by multiplying its response reliability to each odor. For instance, ...

Reviewer 2

We thank the reviewer for their careful reading of the paper and the detailed and perceptive comments on the experimental and modeling aspects, which have helped in improving the paper significantly.

1. *The principal areas that need to be strengthened before publication are:*

   - *Show that unreliable cells actually contribute to model performance at discriminating similar odors*
   - *Establish how much of the observed stochasticity in responses is real neuronal variability vs. arising from experimental noise*
   - *Clarify if APL→KC noise is really the only way the observed stochasticity could arise*

   Thank you for providing the summary. We addressed all three of these points in the response that follows.

2. *The most important conclusion is that unreliable cells allow similar odors to be distinguished because their responses are uncorrelated even for similar odors. However, their only measure here (Fig 6) is discriminability - D(A,B) - which isn't a measure of accurate discrimination per se but rather how different are the output weights of a certain KC toward the avoid vs. approach output neurons. It's not clear if this discriminability measure translates into actual discrimination. Maybe the unreliable cells are too few in number for their superior discriminability to improve the actual discrimination?*

   We agree that our measure of discriminability may not be the full story when it comes to actual discrimination. We added the following text when explaining our reasoning: The actual mechanism for discrimination given KC weights to avoid and approach neurons remains to be demonstrated — e.g., is it subtractive? divisive? We simply assume that the larger the difference in the weights, the easier it is to discriminate, but other mechanisms, e.g., divisive, may exist as well.

   In terms of cell numbers, for any odor the number of highly reliable cells form the smallest percentage of responsive cells. So, the least reliable cells form the largest part of the population, and that is the reason why unreliable cells play a part in discrimination with extended training. Fig. S4 in the paper (and Fig. 5 below) now shows the average number of responsive cells per reliability class.

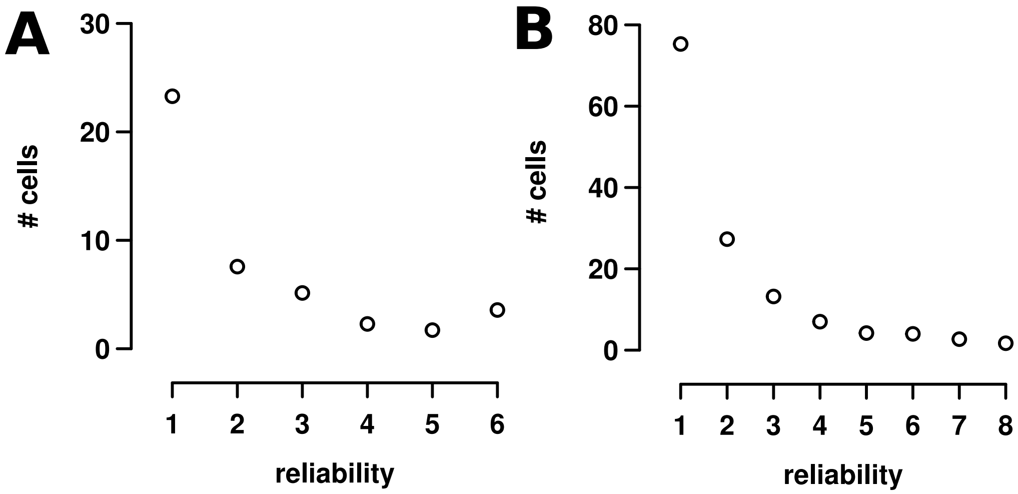

Number of cells in each reliability class for flies and mice. As reliability increases, the number of cells in that reliability class decreases, in both flies (A) and mice (B).

Figure 5: **Number of each class of reliability cells**.

We also examined how much cells in each reliability cell contribute to discriminating similar odors with extended training (Fig. 6, below). This is a drop-out analysis: i.e., we calculate the contribution towards discrimination of each reliability class separately. This approach indicates that low reliability cells contribute strongly to discrimination. It also has the advantage of giving a more graded view of the results, since cells are not simply classified as reliable and unreliable. These plots, shown below as Fig. 6 have been included in the manuscript as Fig. 7E,F.

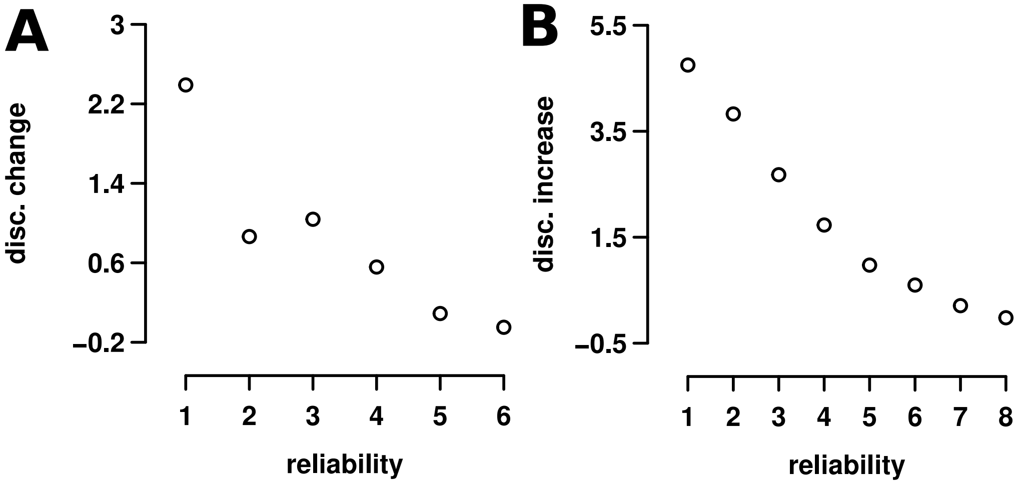

The improvement in discrimination, D, from normal to extended training for similar odor-pairs in flies (A) and mice (B). Each point represents the contribution of cells in a reliability class (x-axis) towards an improvement in the ability to discriminate similar odors with extended training compared to normal training (y-axis).

Figure 6: **Contribution of each class of reliability cells in the ability to discriminate**.

Together, these plots show that unreliable cells play a part in distinguishing similar odors. Even though individually they contribute little, they are much more numerous than reliable cells, and hence can help discriminate with extended training.

3. *To justify their conclusions, the authors need to extend this result to show that a model circuit performs better at learned odor discrimination with unreliable cells than without, and that it performs better when you delete reliable cells, than when you delete unreliable cells. According to the authors' predictions, such a result would only be true for similar odors and only with extended training. This would be like Fig 5E,F, except separating out similar/dissimilar odors, separating out normal/extended training, and using a biological learning algorithm instead of decoder models.*

   Thanks for the suggestion. In the plots shown in Figure 6 (now in Paper Figure 7), for both mice and flies, we show the contribution of each reliability class (on the x-axis) towards separating the odors using the measure D(A,B) (on the y-axis). The population (summed) D for each reliability class was computed after silencing/deleting all cells except those in the chosen reliability class. We then aggregated the computed D scores for all the cells in this category for similar odor-pairs, and that gives the y-axis value. As the plot shows, as reliability increases, their contribution to overall D decreases.

   Also, just to clarify, while we think that unreliable cells are important for discriminating similar odors, unreliable cells may also contribute to discriminating dissimilar odors. We did not stress this point as experiments have clearly shown that the brain can discriminate these odors, with or without the WTA (Lin et al., 2014).

4. *Presumably, the authors believe that experimental artifacts can't explain all of the inter-trial variability. Can they give some justification for an upper bound on how much of the inter-trial variability is experimental noise rather than true neuronal variability (e.g., 5%, 20%, 50%, 100% etc)? This could be based on some reasonable guesstimates of how much photon noise there was, how bad the motion artefacts were, etc. This quantification is important because if the true responses are less variable than the observed responses, then perhaps noise in PNs+PN→KC synapses (Fig 3G) is enough to produce the true variability (no need to invoke noise in APL→KC synapses). It's also important because the stochasticity is the principal experimental result in the paper.*

   About the unreliability of responses: Some of this could come not from neuronal variability, but from signal detection failures or detection noise in the calcium imaging, or the brain moving between trials. Some points to consider:

   (a) *Campbell et al 2013 used GCaMP3 which is much less sensitive than more recent indicators. So the percentages in Fig 1 are probably an underestimate of the true responsiveness.*

   (b) *Presumably there is some threshold for declaring that a cell is responsive or not - but sometimes a cell might genuinely respond but just fall below the analysis threshold*

   (c) *The brain probably moves between trials. They have applied non-rigid motion correction but this probably isn't perfect (and it wouldn't fix z-motion). How sure are the authors that an "unreliable" cell didn't just slightly move outside the ROI during a few of the trials?*

We thank the reviewer for suggesting these appropriate tests. We did various analyses of the data, which we describe below to address the issues that were brought up.

- For flies, as the videos were not archived unfortunately, we tried an alternative method (that likely overestimates noise) to ascertain if unreliable cells emerged due to experimental noise. Specifically, we calculated 'noise' by looking at the fluctuations in dF/F in the pre-stimulus period. Signal variability here includes: i) photon noise, ii) movement noise, and iii) biological noise from spontaneous neural activity activity. So it is likely an overestimate of variability. We then compared that distribution (of 'baseline magnitudes') to the distribution of response magnitudes during odor presentation (Fig. 7 F–I, now in Paper Fig. 1). We observed that less than 10% of KC response magnitudes were within the range of the baseline distribution (Fig. 7G–I). Note that this overlapping set does not mean that the background mean and signal means for the same cell were overlapping. To verify that this was not what was happening, we replotted the background and signal means for the set of cells whose background means overlapped in the distribution plots. Panel I shows this overlapping set of KCs and their background means are less than signal means. Experimental noise is likely a minor contributor to the results here.

  We also show that increasing the threshold for significance, does not change the overall conclusion of the paper about similar odor discrimination (Fig. 8, now in Paper Figure S9) This is consistent with results from electrophysiological recordings of KC odor responses (Turner et al., 2008), which showed qualitatively similar levels of inter-trial variability. We also revised the manuscript to avoid giving the impression there are two distinct classes of cells — reliable and unreliable — and to instead emphasize the spectrum of reliability that is a general feature of sensory representations across a wide range of systems.

  Finally, we want to say a bit more about the imaging analysis pipeline used for this data from (Honegger et al., 2011; Campbell et al., 2013). In the pipeline, we identified in-plane cells by eye, clicked on them to place an ROI, and then a movie of the entire experiment was played underneath this ROI to visually confirm the cell in question did not drift out of plane for the entire course of the experiment. Indeed there were many cells discarded because of movement/possibility of overlap with other cells etc. Typically there were about 200 KCs in a FOV, and our yield of cells was 125 on average. Basically, we dissected a lot of flies to get a reasonable number of high quality preps to analyze.

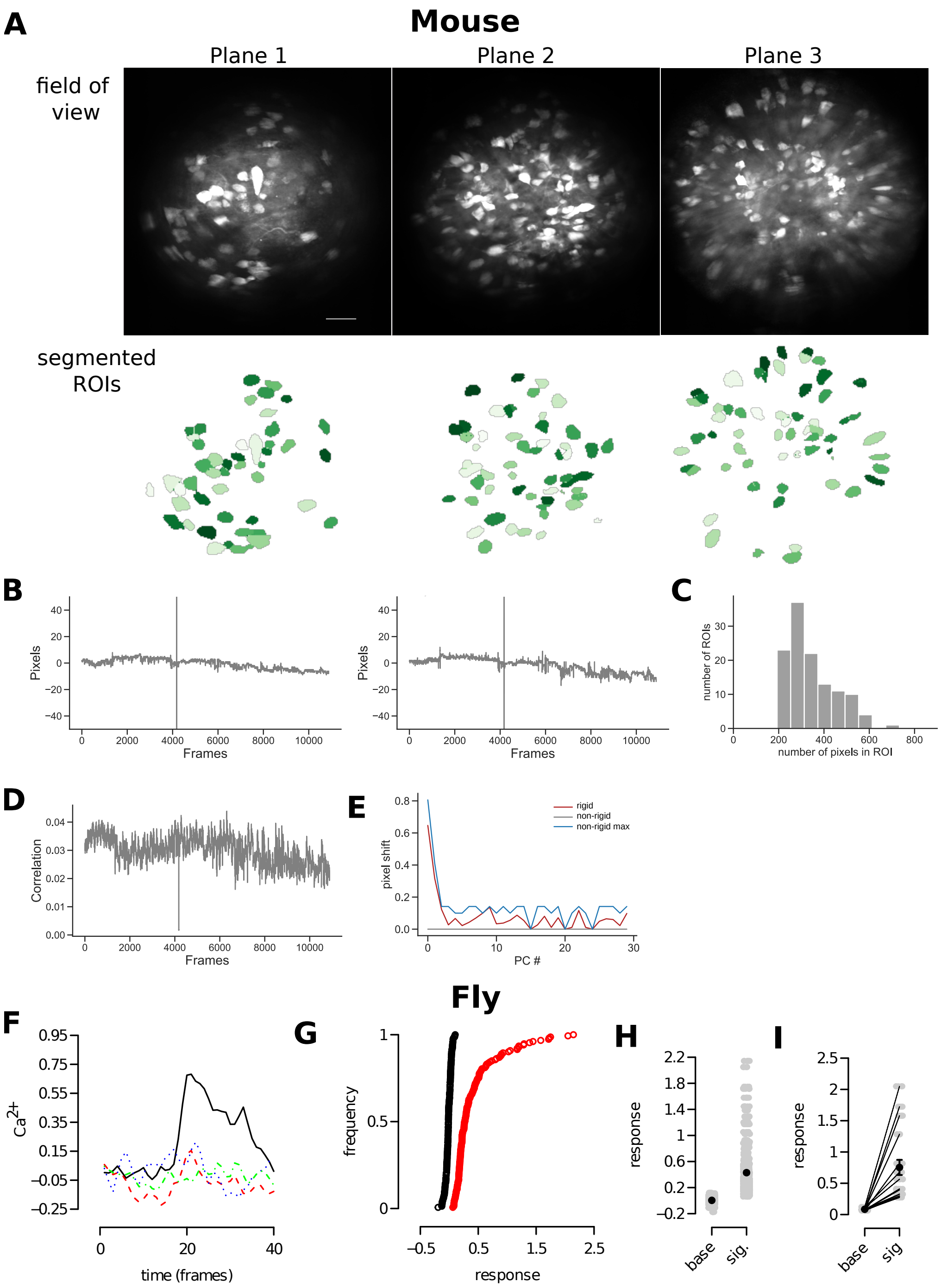

Figure 7: **Detecting signals with calcium imaging.**

(A-E) Mouse calcium imaging data registration and segmentation. (A) Top: Maximum projection GRIN lens field of view of the T-series for each plane imaged. Bar: 100 μm. Bottom: Corresponding region of interests (ROIs) segmented. (B) Pixel shift in the x-axis (left) and y-axis (right) during registration at each represented by frames on the x-axis. (C) Size (in pixels) distribution of each of the segmented ROIs. (D) Peak of phase correlation between frame and reference image at each timepoint. (E) Magnitude of the shifts (both rigid and non-rigid) for each principal component (PC) of the registered movie. (F-I) Fly calcium imaging analysis. (F) A few example traces of different significant cells. (G) A cumulative distribution of the base (black) and signal (red) means. The signal follows a Gamma distribution as we showed in the paper (there is a little bit of a left skew), while the base mean is a very good fit for a Gaussian. (H) Plots of the base (left) and signal (right) means. There are 918 cells in all. The amount of overlap is about 90 cells for both responses. 90 of the base means are above the lowest signal mean response. And, 90 of the signal means are below the highest base mean. We took the overlapping population of cells whose base means were above the lowest signal mean response, and re-plotted their and base and signals means while joining base and signal means for the same cells. We found that in these cells all base means are lower than their signal means.

II We also did a re-analysis of the data to examine how changing the threshold we used to determine if there was a significant response affects the results of the paper. Increasing the response threshold is akin to weeding out cells with weak/questionable responses, i.e., cells with high background means and low signal means. In the paper, the significance p-value threshold was $\alpha = 0.01$. We repeated this analysis for α values 0.005, 0.001, 0.0005 (Fig. 8, below). Increasing the selectivity of what we call a response decreased both the number of reliably- and unreliably-responding cells. This change, however, does not affect the main result of the paper: unreliable cells improve discrimination of similar odors with extended training. Also, when we *decreased* the selectivity of our response criterion ($\alpha = 0.05$) unreliable cells still improved discrimination, but to a lesser extent.

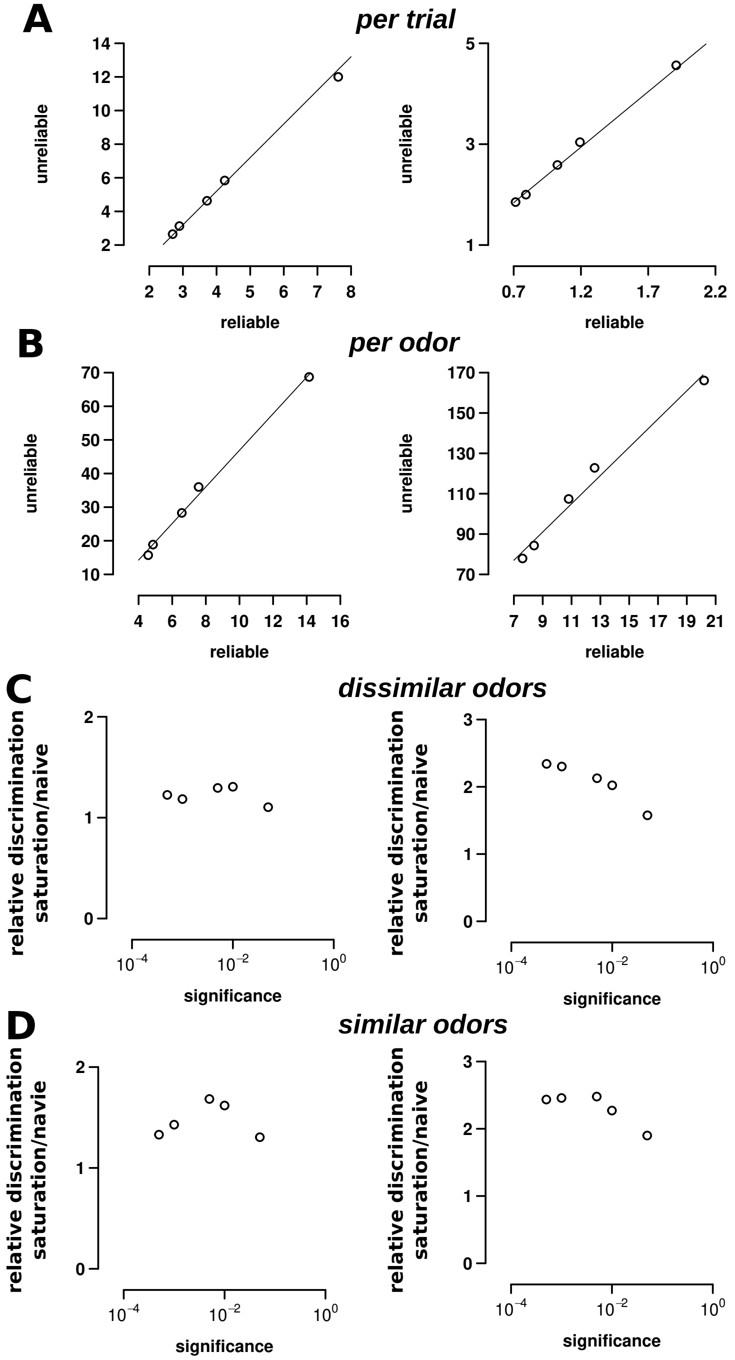

Figure 8: **Effect of changing thresholds on cell reliabilities and discrimination**. The left column is fly data and the right column is mouse data. (A) Changes in the number of responsive reliable vs. unreliable cells per trial with change in alpha, the significance level. alpha increases from 0.0005 on the left to 0.05 on the right; the alpha in the main figures is 0.01. As alpha increases, more cells respond, and the increase in reliable and unreliable cells is proportional. (B) Changes in reliable vs. unreliable cells per odor. Again, the increase in number of cells with alpha is proportional. (C, D) Relative discrimination contributions of unreliable and reliable cells with learning (saturation) vs. without learning (naive) plotted against significance, for dissimilar odors. Changing alpha does not significantly change discrimination ability. For both C and D changing alpha also does not change the relative discrimination ability between dissimilar and similar odors.

As we decreased response criterion selectivity further ($\alpha = 0.1$, Fig. 9 below, also Paper Fig. S9), we began to see the consequences of essentially classifying every cell as reliable. The proportion of reliable/unreliable cells skewed towards 'reliable', and consequently the contribution of unreliable cells to discrimination began to drop off, while contributions of reliable cells increased. Unsurprisingly, with normal training, where most of the contributions come from reliable cells, we observed a marked increase in similar odor discrimination ability, so much so that similar odor discrimination was better than dissimilar odor discrimination: a result that is not borne out by experiments. This suggests that $\alpha = 0.1$ is unlikely to apply here as a significance measure.

Thus, this analysis suggests that reasonable variations in the significance threshold does not affect the result of the paper, and that extended training improves discrimination because of unreliable cell contributions.

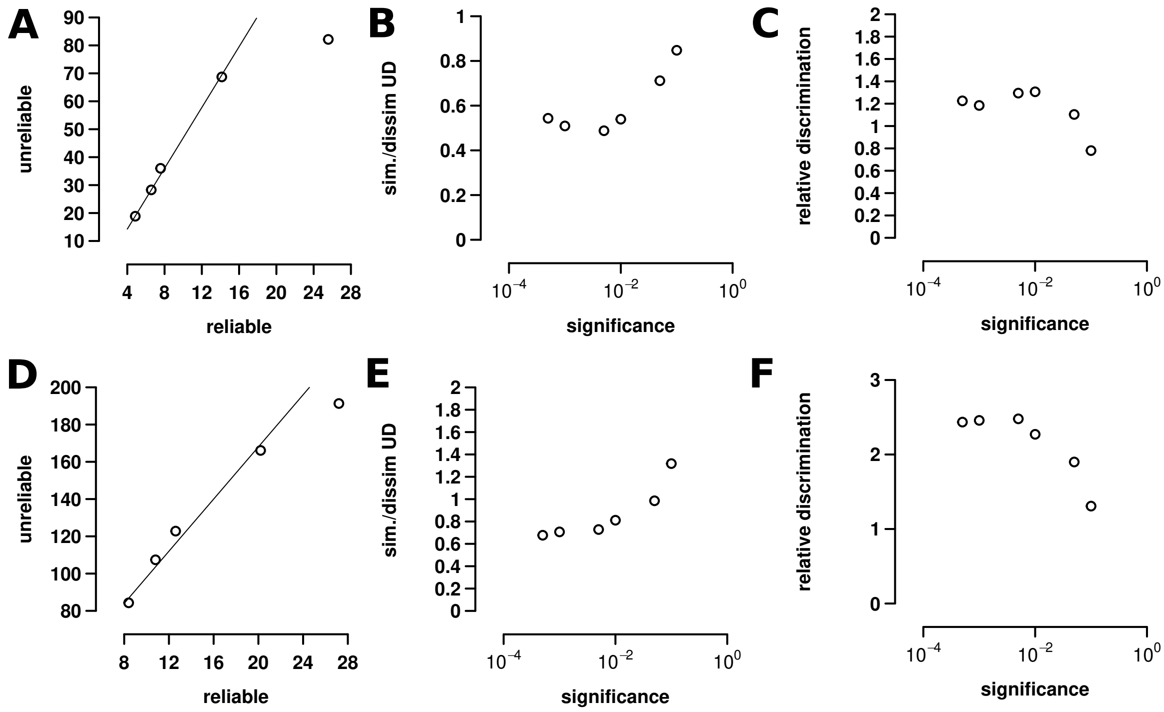

The effect of the significance level on cell responses and discrimination.
(A) With an increase in alpha (p = 0.005, 0.01 etc.), the number of cells increases, increasing the number of reliable and unreliable cells. For (p = 0.1), the increase in reliable cells, however, is a lot more than the increase in unreliable cells because there is a finite pool of cells that are available. p = 0.1 nearly exhausts all the cells, and given the threshold, a lot of them become reliable, and there aren't enough cells that can become unreliable.
(B) Here, we plot discrimination quotient for similar vs. dissimilar odors with normal training. With lower alphas, wherein very few cells are reliable, the difference is big. The unreliable cells only come into play with later training. With alpha=0.1, however, now there are difference between reliable cells themselves. What were formerly unreliable cells are reliable, and they respond differently in terms of amplitude. So, even the similar odor quotient is high, indicating that similar odor discrimination should be good right off the bat.
(C) This is the increase in discriminatory power with extended training. Again, for alphas < 0.1, there is an increase in discriminatory power. What this means is that for similar odors, there is an improvement in discrimination. For alpha=0.1 for flies, there is a decrease to around 0.8.
(D-F) Same figures, except for mice this time.

Figure 9: **Effect of varying the significance threshold. Top row: fly data, bottom row, mouse data**.

III One concern that the reviewer highlighted was that the cells might move, and this movement might contribute to reliable cells being classified as unreliable. We have included a video of the subset of the mouse cell population for the entirety of the experiment, i.e., all odor exposures. In the video, we have outlined the cells using the segmentation software that was used for identifying cells and getting their intensity originally. The outlines clearly show that the cells do not move out of the segmented outlines significantly even when the brain moves. The plots in the accompanying figure (Fig. 7) show the total amount of movement in the x and y axes, and again this movement is negligible compared to the area of the cell. The distribution of cell sizes below shows that cell sizes range between 200-600 pixels, clearly an order of magnitude higher than the x and y movements.

We have also attached two videos. They can be accessed at:

```
Mouse164_Plane_2.mov is a speeded up video of the experiment:
https://snl.salk.edu/~shyam/Mouse164_Plane_2.mov
```

Mouse164_Plane_2_ROIS.mov is the same video, but with the ROI segmentation.
https://snl.salk.edu/~shyam/Mouse164_Plane_2_ROIS.mov

5. *About the role played by noise in APL-KC synapses (Fig 3): It seems the principal role is by making the effective spiking threshold of a KC vary from trial to trial - sometimes the excitation from a particular odor is enough to make the KC spike, sometimes not. Couldn't this arise not (only) from noise in APL→KC synapses, but rather (or also) from noise in the integration of synaptic excitation? - see Groschner et al 2018 Cell - the subthreshold depolarisation and then spiking (or not) of KCs certainly seems very noisy and variable across trials. (Of course, the measurements in Groschner include noise from both excitation and inhibition, but the point is we can't tell which one it is.) I couldn't tell from the Methods, but I suspect that in the rate coding model, inhibition and thresholding both take the same form (subtraction) - or perhaps are even literally the same thing since I don't see mention of a separate thresholding step. (Note that noise in KC integration would be a distinct source of noise from noise in PN→KC synaptic strength, which the authors argue is not sufficient to produce observed unreliability. The difference seems to be that noise in PN→KC synaptic strength gets averaged out across the many PN inputs for each KC, but noise in the KC's intrinsic properties doesn't.) I don't think the authors can conclude that only APL-KC noise (rather than noise in KC synaptic integration in general) can explain the observed stochasticity.*

   The reviewer brings up a good point, and we appreciate the reference to Groschner et al. We added KC noise as one of the parameters for noise exploration. We found (Paper Fig. 4 and S10) that KC noise by itself doesn't lead to a stochastic code, however, it reduces the amount of noise needed in APL to generate the stochastic code. In the updated manuscript, we have toned down our earlier claim, and instead say that WTA is the best candidate for generating a stochastic code, but, in order to work, it requires noise in other components, too. Additionally, we acknowledge that new mechanisms may emerge as we find out more about the connectome, hence our reason to say "candidate". We thank the reviewer for the reference and the possibility of KC noise, whose inclusion has improved the model. Please refer to the Modeling and Theory section of the paper for all the changes that we made to the previous model. We also discuss some of these changes in See R1#10 of this document.

6. *Another major conclusion is that unreliable cells have less correlated responses to odors (Fig 4). I'm having trouble evaluating the conceptual novelty of this conclusion. Could it simply be mechanically true that when any cells respond unreliably, they're less likely to overlap for different odors, almost by definition? If every cell responds completely randomly, would you still expect that cells that respond more often would have more overlap than cells than respond less often? Similarly, for Fig 4C, could it simply be that unreliable cells are responding rather randomly, thus the population responses of unreliable cells are uncorrelated because the responses are pretty much random? But perhaps this is precisely the authors' point? They should clarify whether they interpret this result as "unreliable cells must inevitably be less overlapping, by definition" or "it's not necessarily true that unreliable cells are less overlapping, but they are in this particular dataset, i.e. it's a feature of fly/mouse coding schemes that unreliable cells are less overlapping". They could perhaps demonstrate which of these is the correct interpretation by creating fictitious random responses not based on real olfactory inputs, and showing that the unreliable cells are/aren't less overlapping.*

   The notion that unreliable cells are less likely to overlap for different odors is only partly true by definition. A cell's reliability is assigned based on its response frequency to a single odor. This means that a cell that is unreliable for odor A could be reliable for odor B. We did not make this clear originally, but in Paper Fig. 5C, there is one data point for the pair (A,B) and another data point for the pair (B,A) — i.e., the overlap measure is not symmetric. For the former, we find each unreliable cell for odor A and multiply its response frequency by its response frequency to odor B. This means that the overlap of an "unreliable cell" could be as high as 3/6 x 6/6 = 0.50, or as low as 1/6 x 0/6 = 0. Similarly, the overlap of a "reliable" cell could be as high as 6/6 x 6/6 = 1.0, or as low as 4/6 x 0/6 = 0 with intermediate values like 4/6 x 1/6 = 0.083. Thus, while there is some bias for reliable cells to have more overlap than unreliable cells, it is not entirely true by definition.

   To make this clearer in the manuscript, we added the following text: We then split KCs into reliable and unreliable classes and tested how well each class preserved odor similarity. For the former, for odor pair (A,B), we found the set of reliable KCs for odor A, and computed their correlation with responses to odor B. Importantly, if a KC is reliable for odor A, it may be reliable or unreliable for odor B, and thus this similarity measure is not symmetric. In the figures that analyze reliable and unreliable KCs separately, there is one dot for odor pair (A,B), and another dot for pair (B,A). While there is some bias for unreliable cells to be less overlapping than reliable cells — simply because unreliable cells respond less frequently — it is not inevitable since an unreliable cell could be reliable for others odors, and vice-versa. Note, that in Fig. S4 of the paper we show that less reliable cells are not completely random, they still have some level of odor selectivity or bias. We also elaborate on the bias of reliable and unreliable cells in R1#20 of this document.

7. *Fig 4-6: the authors split cells into reliable and unreliable classes. But as far as I understood from Fig 1 and p.36, reliability is only defined relative to a specific odor. What do they do for cells that are reliable to odor A but unreliable*

to odor B? Are these excluded from analysis? Also, it's not clear to me for Fig 4B,C that it's valid to draw a scatter plot and trend line when every dot represents a different set of KCs (since they represent different odor pairs that must have a different set of reliable and unreliable KCs) - the authors need to be more convincing of the rationale for this analysis.

This relates to our response to above re: the non-symmetry of the overlap measure. We did not exclude any cells from the analysis, but rather in one test (A,B) the cell may be placed into the "reliable" class, and in the other test (B,A), the cell may be placed into the "unreliable" class.

Re: trend lines — Similar trend lines were drawn in other plots where dots represented different KCs (e.g., Paper Fig. 2E, Fig. 3B, etc). Thus, it is not clear to us why a trend line is invalid when the set of cells are different across dots.

8. *Related to this, for Fig 4D-G: How is overlap affected by whether a neuron has the same vs. different reliabilities for A vs. B? (Is this a straightforward result of the definition of overlap =P(A)*P(B) as per my point #4 above or is it more complicated?) Fig 4D-G - Shouldn't overlap probability be the y-axis, and reliability the x-axis? It seems like their argument is that overlap depends on reliability, not that reliability depends on overlap. The way they describe "slope steepness" in the text also seems to require swapping the x and y axes.*

   Yes. The overlap is the definition of P(A)*P(B). As we mentioned above, overlap depends on the odor being considered. If it is A vs. B, and the cell responds in 1/6 trials for A and 4/6 trials for B, then overlap(A,B) would be in the reliability=1 class vs. reliability=4 for overlap(B,A).

   Re: axes swapping — We originally plotted it this way as we felt that the relation between overlap and reliability for similar odors came out more easily. But, we see the reviewer's point, and have changed the axes. Thanks for the suggestion.

9. *I don't understand the conclusions drawn from Fig 5A,C and Fig S8. - "The top 25% of cells are more similar than the rest of the population (i.e., they provide no extra discriminatory information)." - I don't see how the parenthetical follows from the first part of the sentence - Fig S8 title "Sparse coding does not improve discrimination ability for similar odors." I don't see how this follows from the figure - "we found that the cosine similarity of odors did not decrease from the top 50% of responsive cells to the top 25% of cells for similar odors (top circles), which would be expected if sparse coding improves discrimination." I don't think the first part of the sentence is a common prediction of the idea that sparse coding improves discrimination - the latter idea is usually that if few cells are active in general, then two different odors would likely have relatively few overlapping cells - it's not that the strongest-responding cells are supposed to form the most distinctive patterns. Moreover, cosine similarity doesn't necessarily correspond to the accuracy of discrimination (this comes back to my point #1).*

   Apologies for the confusion. We changed the first sentence to read: The top 25% most responsive cells are more similar than the responses of cells in other percentiles. In other words, the top 25% most responsive cells have the highest overlap for similar odors, and thus they provide less discriminatory power, compared to cells in other percentiles.

   We changed the second sentence to read: Cells with high responses demonstrate the lowest overlap for dissimilar odors, but the highest overlap for similar odors. Then, to bring in the sparse coding aspect, we added to the caption: Sparse coding methods typically retain only the highest responding neurons (here, the top 25% of cells). Thus, these results challenge whether sparse coding alone is sufficient for discriminating similar odors.

   For the third sentence, we agree that this is an important distinction to make. Most would agree that sparse coding would likely generate non-overlapping representations for different odors. But for similar odors, the role of sparse coding is less clear. Computationally according to some models, the sparser the representation is, the less likely that even similar odors would overlap, since sparsity is believed to create distinction. On the other hand, if the strongest-responding cells represent the "dominant" pattern present in the inputs, then these cells are likely to overlap if odors are very similar. The latter perspective is largely our point (which the reviewer also made), but we don't think this is a very common view of sparse coding. It also leads to questions about what the optimal trade-offs are between the level of sparsity (higher storage capacity) and the discrimination power for similar odors. We discuss this later in the Discussion ("Benefits of stochastic coding") where we suggest that unreliable cells may encode more subtle features, or higher order odor statistics, that are useful for discrimination. To make this clearer in the text, we changed this sentence to read: We found that the cosine similarity of odors did not decrease from the top 50% of responsive cells to the top 25% of cells for similar odors (top circles), which would be expected under models that assume that sparser codes lead to less representational overlap and more distinction.

   We agree that cosine similarity may not reflect the accuracy of discrimination; more generally, how KCs or PCx neurons are "read-out" by downstream neurons is not completely clear. We simply assumed that the most similar the responses, the harder discrimination would be — i.e., we hope that cosine similarity correlates with whatever is the true discrimination mechanism — but we agree this was not made clear. So, we added: We use cosine similarity as a

proxy for how difficult it may be for downstream read-out mechanisms to distinguish odors (higher cosine similarity→ more difficult discrimination, since it remains unclear how exactly representations are read-out to drive behavior).

10. *"As the reliability of individual KCs increased, their mean response levels increased exponentially" - I'm not sure this is the best way to describe it. In both mice and flies, the last point (100% reliability) is clearly an outlier and one would also think a priori that it would be an outlier. The first few points actually seem to increase linearly - which is actually what you'd expect purely mechanically if the cells have the same response when they do response, but when this is averaged with the non responses, the mean response across trials is lower for the more unreliable cells. If there's a break between two different trends, this actually supports the idea of a qualitative difference between reliable and unreliable cells. (I'm not sure this point is actually that important. The exponential fit seems to me to have no relevance to the final conclusions and the exponential relation hints at some kind of deeper mathematical truth which I suspect isn't real - I suggest just removing this, unless I've missed something.)*

    First, to clarify one point: the averaged response is obtained by only considering those trials in which the cell had a significant response. Thanks to this suggestion, we now make this explicit: The averaged response is obtained by only considering those trials in which the cell had a significant response.

    With regards to the exponential fit, we acknowledge that it might not be significant. One the other hand, it gives a very simple way (two parameters) to describe the data. However, whether the last point is an outlier might require more analysis, and, as suggested, we have taken it out.

11. *Analysis of data from Lin et al 2014 - Fig S7D - x-axis says "firing rates" but the data from Lin et al 2014 was calcium imaging - where do the firing rates come from?*

    Yes, the reviewer is right. We have changed it. Thank you for pointing out the error.

12. *In the model in Fig 3 the authors show how the models can recover the correct ratio of reliable:unreliable cells and other aspects from Fig. 2. It would be helpful to show (perhaps in a supplemental figure) if the models reproduce other aspects of the imaging data, e.g. the CDF of reliability, response amplitude, probability of overlap as in Fig. 2. Do they also follow a gamma distribution? Since these are curves rather than single metrics, it wouldn't be practical to show this for every model, but they could show some example curves (e.g., their favorite best-fitting model plus some that fit badly). Or if every model fits a gamma distribution, perhaps they could plot the gamma fit parameters as the y-axis of graphs akin to the graphs in Fig 3C-G. This would show both that the models are accurate, and that the reliability statistics don't depend on the number of cells (124 KCs in the data, X KCs in the model)*

    Thanks, these are great suggestions. In response, we added several new analyses. First, we picked our favorite best-fitting model and one poorly performing model and plotted their response fit for a Gamma distribution in the supplement (Fig. S11E). Second, we present a better description of the successful parameters in Methods. Third, we plotted how discrimination improves when the responses are evaluated with a learning model. Fourth, we like the suggestion of independence of the results from the number of KCs, and give an example of that, too (Fig. S11A–D). Fig. S11A,E also contain results for discrimination improvements with extended training similar to Paper Fig. 7. These figures also serve as another successful parameter combination set, and include characteristics of the code such as the distributions for reliability, responses and overlap.

    We added the following to the modeling section: As we show in Figure S11, "successful" parameter combinations also recapitulated other observed features, including response-reliability relationships (Paper Fig. 2E), a Gamma distribution of response magnitudes (Paper Fig. 2C), and discrimination characteristics discussed later (Paper Fig. 7). Additionally, Figures S11A–D we show that our results are independent of MB size.

13. *There is some confusing/missing information in the Methods:*

    (a) *For the rate coding model in Fig. 3, the authors refer to their previous publications but don't provide the equations here or other details like the # of KCs. To save the reader having to track back into previous papers, they should provide the relevant equations and details here. The equations are important to understand the parameter values given in Table S1*

    (b) *The Methods provide contradictory information about how many claws were assigned to each KC - a Poisson distribution (p. 37, first paragraph under "Figure 3") or a Binomial distribution (p. 38, last paragraph before "Figure S7"). Which is it? And what is the justification for using these distributions - can they be fit to real data? I could not find the analysis in Sterling and Laughlin 2015 explaining how parameters were fit to claw numbers of PN noise (indeed Caron et al 2013 and Bhandawat et al 2007 don't seem to be cited in Sterling and Laughlin 2015)*

    Thank you for pointing this out. The Sterling reference was made accidentally; we meant to cite Stevens (2015). This is now fixed. We also added the Equations, as suggested, to the modelling and theory section in Methods.

    Briefly, Stevens (2015) analyzed Caron et al. (2013); Bhandawat et al. (2007); Gruntman & Turner (2013) and provided the underlying distributions for the data. The number of Kenyon cell claws are chosen from a Binomial distribution with

a mean of 8 and probability of success of 0.715 (Caron et al., 2013). The synapse distribution was taken from a Gamma distribution with shape and scale parameters of 4 (Gruntman & Turner, 2013). The glomerular response distribution uses an exponential distribution (Bhandawat et al., 2007).

14. *Some points the authors might wish to consider for the Discussion - on the "saturate the cloud" vs "move the cloud" strategies, the authors should consider Baltruschat et al 2021 eLife which suggests that training leading to long-term memory actually can change KC representations. So there could be "cloud moving" in flies too - but only with repeated training - consistent with the results from Chapuis and Wilson - The authors may wish to discuss their results in the context of the discrimination vs. generalisation trade-off (eg Barak et al 2013 which the authors have already cited) - It's probably good for similar odors to activate the same reliable cells because this allows the animal to generalise to similar odors (which could actually be the same odor asthe trained odor but with some sensory noise added).*

    We thank the reviewer for the references. We added the following to Discussion: The third model moves the clouds apart via the addition of new cells (colored in yellow) that were not previously responsive to the odor. In addition, unreliable cells may become more reliable with extended training. Recent work by Baltruschat et al. (2021) demonstrated that repeated training leading to long-term memory can change KC representations in flies. Further, in mice, representational drift (Schoonover et al., 2021) is another form of "moving the cloud", albeit over longer time-scales and in the absence of training.

    To mention generalization, we added: However, given two similar odors, we showed that there is stronger correlation in the activities of reliable cells than the activities of unreliable cells (Figs. 3,5). Reliable cells could be advantageous if the goal of the animal is to generalize behaviors from one odor to another similar odor. On the other hand, unreliable cells may encode important information ... [to distinguish odors]

Minor points

1. *Fig S5 B, E - how can there be probabilities > 1? Perhaps I misunderstood these graphs – can the authors clarify?*

    Fitting the cumulatives gives the parameters of the Gamma distribution for flies as shape and scale factors of 0.15 and 0.44 for dissimilar odors, and 0.21 and 0.67 for similar odors. The Gamma distribution for mouse is defined by shape and scale factors of 0.24 and 0.09 for dissimilar odors, and 0.22 and 0.45 for similar odors. Probabilities are not < 1, but sometimes probability density functions can have values greater than 1. The important thing is that, when integrated, the function sums to 1. In this case, if you consider the range of integration it is 1. The minimum point for flies is 0.028 and for mice it is 0.156.

2. *"Indeed, when examined over all trials (Fig. 1D), a total of 29% of the 124 KCs were unreliable, whereas 6% of the 124 KCs were reliable", which, as expected, is similar to the 5% fraction on an average trial."Would this be clearer if they say "examined over all trials of each odor"?*

    Thanks! We made the change to the second paragraph of the first section of results.

3. *Fig 2 – gamma distribution – is this including only the cells that respond at least once? (i.e., ignores the silent cells?)*

    Yes, the silent cells are left out in this calculation. We make this point in methods now.

4. *p. 36 - "When we are considering odor-pair AB, the cell's overlap would be the reliability of the cell for odor A." - surely this is only true when the cell's reliability for odor B is 100%? Or is this a typo and it's supposed to say "the cell's *reliability* would be the reliability of the cell for odor A."?*

    Thanks for pointing out the typo. The text is now, " the cell's reliability would be reliability of the cell for odor A"

5. *p. 4 "cell 3 with a reliability of 1" suggest "reliability of 1 out of 4"? "1" creates momentary confusion, sounds like a perfect score of 1.0*

    Good point. Now changed as suggested.

6. *Fig 7 - the arrows are a bit hard to distinguish thickness. Could they make the thick lines thicker?*

    We made them thicker. Appreciate the tip.

7. *Fig S5 - consider labelling the red and black curves directly on the figure*

    Now, annotated. Thanks.

8. *Fig 3H - the graded color bar on the right doesn't show up as graded on the MacOS Preview App (but it's OK on Adobe Acrobat) - might want to double check this before publication.*

    Thank you for the heads up. We will preview it on different programs to see how it comes out. Also, this is no longer part of the main figure.

9. *Fig 3H legend "Darker colors indicate lower amounts of noise" - should this be "higher".*

   Thanks for pointing that out. Fixed now. Fixed in legend S7D

10. Fig 4B,C - should the y-axis labels say "KC similarity score" rather than "reliable KCs" and "unreliable KCs"?

    Yes. We have changed it to say this now.

11. Citations: - Given the use of both mouse and fly, perhaps the authors would like to cite this recent review comparing the two systems? Endo and Kazama 2022 Curr Opin Neurobio - one of the bioRxiv preprints cited has now been published (Abdelrahman et al 2021 PNAS)

    Thank you for the update and suggesting Endo and Kazama. We have updated the Abdelrahman reference, and mentioned the second reference in discussion and results.

12. Typos p. 8 "Although, it does not capture a neuron's inner workings" - delete comma p. 18 "other studies have shown at least three ways in which variability can improve an animal's fitness." - is this supposed to be "two ways"? the following text says (1) variability expands the search space, (2) stochastic resonance, with the authors' new idea of improving discrimination as the third way. Missing equation number ?? on page 39, Figure 6. p. 10 "In other words, as overlap increases (x-axis in Figs. 4E-F), the reliability (y- axis) increases proportionally: fly: r = 0.87, mouse: r = 0.91. Conversely, for dissimilar odor pairs (Figs. 4G-H), as overlap" - the panel references seem to be off by one? Same on p. 36 ("Figure 4E-H" - should be D-G)

    Thanks for catching these. We changed all of these as suggested.

References

Abdelrahman, N. Y., Vasilaki, E., & Lin, A. C. (2021). Compensatory variability in network parameters enhances memory performance in the drosophila mushroom body. Proceedings of the National Academy of Sciences, 118, e2102158118.

Amin, H., Apostolopoulou, A. A., Suárez-Grimalt, R., Vrontou, E., & Lin, A. C. (2020). Localized inhibition in the drosophila mushroom body. Elife, 9, e56954.

Baltruschat, L., Prisco, L., Ranft, P., Lauritzen, J. S., Fiala, A., Bock, D. D., & Tavosanis, G. (2021). Circuit reorganization in the drosophila mushroom body calyx accompanies memory consolidation. Cell reports, 34, 108871.

Bhandawat, V., Olsen, S. R., Gouwens, N. W., Schlief, M. L., & Wilson, R. I. (2007). Sensory processing in the drosophila antennal lobe increases reliability and separability of ensemble odor representations. Nature neuroscience, 10, 1474–1482.

Campbell, R. A., Honegger, K. S., Qin, H., Li, W., Demir, E., & Turner, G. C. (2013). Imaging a population code for odor identity in the drosophila mushroom body. The Journal of Neuroscience, 33, 10568–10581.

Canto-Bustos, M., Friason, F. K., Bassi, C., & Oswald, A.-M. M. (2022). Disinhibitory circuitry gates associative synaptic plasticity in olfactory cortex. Journal of Neuroscience, 42, 2942–2950.

Caron, S. J., Ruta, V., Abbott, L. F., & Axel, R. (2013). Random convergence of olfactory inputs in the drosophila mushroom body. Nature, 497, 113–7.

Gruntman, E. & Turner, G. C. (2013). Integration of the olfactory code across dendritic claws of single mushroom body neurons. Nature neuroscience, 16, 1821–1829.

Haberly, L. B. (2001). Parallel-distributed processing in olfactory cortex: new insights from morphological and physiological analysis of neuronal circuitry. Chemical senses, 26, 551–576.

Honegger, K. S., Campbell, R. A., & Turner, G. C. (2011). Cellular-resolution population imaging reveals robust sparse coding in the drosophila mushroom body. The Journal of Neuroscience, 31, 11772–11785.

Johnston, R. J., Chang, S., Etchberger, J. F., Ortiz, C. O., & Hobert, O. (2005). Micrornas acting in a double-negative feedback loop to control a neuronal cell fate decision. Proceedings of the National Academy of Sciences of the United States of America, 102, 12449–12454. 961QC Times Cited:115 Cited References Count:28.

Li, F., Lindsey, J. W., Marin, E. C., Otto, N., Dreher, M., Dempsey, G., Stark, I., Bates, A. S., Pleijzier, M. W., Schlegel, P., Nern, A., Takemura, S.-y., Eckstein, N., Yang, T., Francis, A., Braun, A., Parekh, R., Costa, M., Scheffer, L. K., Aso, Y., Jefferis, G. S., Abbott, L. F., Litwin-Kumar, A., Waddell, S., & Rubin, G. M. (2020). The connectome of the adult drosophila mushroom body provides insights into function. eLife, 9, e62576.

Lin, A. C., Bygrave, A. M., de Calignon, A., Lee, T., & Miesenbøck, G. (2014). Sparse, decorrelated odor coding in the mushroom body enhances learned odor discrimination. Nature neuroscience, 17, 559–568.

Pouget, A., Beck, J. M., Ma, W. J., & Latham, P. E. (2013). Probabilistic brains: knowns and unknowns. Nature neuroscience, 16, 1170–1178.

Prisco, L., Deimel, S. H., Yeliseyeva, H., Fiala, A., & Tavosanis, G. (2021). The anterior paired lateral neuron normalizes odour-evoked activity in the drosophila mushroom body calyx. Elife, 10, e74172.

Roland, B., Deneux, T., Franks, K. M., Bathellier, B., & Fleischmann, A. (2017). Odor identity coding by distributed ensembles of neurons in the mouse olfactory cortex. Elife, 6.

Rule, M. E., OÂ¿Leary, T., & Harvey, C. D. (2019). Causes and consequences of representational drift. Current opinion in neurobiology, 58, 141–147.

Schoonover, C. E., Ohashi, S. N., Axel, R., & Fink, A. J. (2021). Representational drift in primary olfactory cortex. Nature, pp. 1–6.

Stettler, D. D. & Axel, R. (2009). Representations of odor in the piriform cortex. Neuron, 63, 854–64.

Stevens, C. F. (2015). What the fly's nose tells the fly's brain. Proceedings of the National Academy of Sciences, 112, 9460–9465.

Turner, G. C., Bazhenov, M., & Laurent, G. (2008). Olfactory representations by drosophila mushroom body neurons. Journal of neurophysiology, 99, 734–746.

Woods, N. I., Stefanini, F., Apodaca-Montano, D. L., Tan, I. M., Biane, J. S., & Kheirbek, M. A. (2020). The dentate gyrus classifies cortical representations of learned stimuli. Neuron, 107, 173–184.
