# Supplementary material for: Effects of stochastic coding on olfactory discrimination in flies and mice
Source: PLoS Biol. 2023 Oct 31;21(10):e3002206. doi: 10.1371/journal.pbio.3002206 (PMC10618007; doi:10.1371/journal.pbio.3002206)
Supplement: Attachment — Submitted filename: second_reviewer_response.pdf [file pbio.3002206.s017.pdf]

Stochastic coding paper reviewer response: Part II

We are happy that we were able to address both reviewers concerns about the paper. We briefly outline what we did to address the minor points of the second reviewer.

Reviewer 2:

1. Can the authors put a dotted line on graphs to indicate the 0 line or change the tick marks on the axes so that there's a tick mark at 0? (e.g. Fig 1F y-axis, 1G x-axis, Fig 5A-C, 7E,F)

   We thank the reviewer for helpful suggestions on making the plots more clear. As the reviewer requested, we have now included a 0-line. Fig. 1 is now Fig. 8. We have made changes to Figures 3C, 4A–C, 6E–F, 8F–H. We also explain the 0-line in the legends.

2. Some y-axes need to be changed to start at 0, e.g. Fig 6A,

   Thank you for the suggestion. We made changes to Fig. 5A,C to make the axes start from 0.
